# Engineering receptors in the secretory pathway for orthogonal signalling control

**DOI:** 10.1038/s41467-022-35161-0

**Published:** 2022-11-29

**Authors:** Mohamed Mahameed, Pengli Wang, Shuai Xue, Martin Fussenegger

**Affiliations:** 1 Department of Biosystems Science and Engineering, ETH Zurich, Mattenstrasse 26, CH-4058 Basel, Switzerland; 2 Faculty of Life Science, University of Basel, Mattenstrasse 26, CH-4058 Basel, Switzerland

**Keywords:** Synthetic biology, Genetic engineering

## Abstract

Synthetic receptors targeted to the secretory pathway often fail to exhibit the expected activity due to post-translational modifications (PTMs) and/or improper folding. Here, we engineered synthetic receptors that reside in the cytoplasm, inside the endoplasmic reticulum (ER), or on the plasma membrane through orientation adjustment of the receptor parts and by elimination of dysfunctional PTMs sites. The cytoplasmic receptors consist of split-TEVp domains that reconstitute an active protease through chemically-induced dimerization (CID) that is triggered by rapamycin, abscisic acid, or gibberellin. Inside the ER, however, some of these receptors were non-functional, but their activity was restored by mutagenesis of cysteine and asparagine, residues that are typically associated with PTMs. Finally, we engineered orthogonal chemically activated cell-surface receptors (OCARs) consisting of the Notch1 transmembrane domain fused to cytoplasmic tTA and extracellular CID domains. Mutagenesis of cysteine residues in CID domains afforded functional OCARs which enabled fine-tuning of orthogonal signalling in mammalian cells.

## Abstract (web summary)

Artificial receptors targeted to the secretory pathway often fail to exhibit the expected activity due to post-translational modifications and/or improper folding. Here, the authors engineer diverse synthetic receptors that reside in the cytoplasm, inside the endoplasmic reticulum, or on the plasma membrane through orientation adjustment of the receptor parts and by elimination of dysfunctional PTMs sites.

## Introduction

Naturally existing receptors are powerful tools in bioengineering settings, but their application is restricted to specific ligands^1–3^. Furthermore, receptors for many clinically important ligands and disease biomarkers have not yet been identified, and in some cases may not exist. Thus, there is a need for artificial receptors that are capable of detecting and responding to user-defined signal inputs^4–7^. In addition to their unique recognition capabilities, such customized receptors can be molecularly coupled to cellular signaling circuits, and indeed, have been extensively utilized in cell-based therapy, diagnostics, and basic research^8^. An example of a successful clinical application of synthetic receptors is the introduction of FDA-approved chimeric antigen receptor (CAR)-T cells^9^. These genetically engineered T cells are equipped with an artificial receptor that enables selective recognition of certain onco-markers presented on malignant cells, facilitating the effective elimination of the target tumors.

Another limitation in the application of natural receptors is that their signaling mechanisms rely on endogenous signaling pathways. These pathways are highly interconnected biochemical reaction networks, and many of them actually share the same components. Therefore, some activatory signaling inputs, especially those that circulate through the whole organism via the blood, can simultaneously act on multiple pathways, leading to unspecific and unexpected cellular outputs^10–12^. To avoid such off-target effects, orthogonal signaling modalities that are independent of endogenous pathways may be preferable due to their highly selective and predictable modes of action^13–15^.

In recent years, different engineering approaches have been employed to design orthogonal receptors for efficient cell signaling control^8,16–18^. One of the main strategies is repurposing molecular components from different parts of known proteins and/or from different species. However, a major challenge is that some of these components do not function as expected when ectopically expressed in mammalian cells. The majority of proteins required proper folding to form the specific three-dimensional shapes necessary for their activity, and this may require the assistance of species- and/or organelle-specific enzymes and chaperones^19–23^. Thus, the same primary polypeptide sequence may be folded in different ways when expressed in different species or even in different cellular organelles of a single species.

The endoplasmic reticulum (ER) is the first destination of proteins following their translocation into the secretory pathway^24,25^. Many such proteins undergo several types of post-translational modifications (PTMs) mediated by ER-resident enzymes. For example, covalent disulfide bond formation between two distantly located cysteine residues, N-linked glycosylation, and O-linked glycosylation are among the major PTMs that occur in the ER^26–28^. Following their transport from the ER, these PTMs may be further modified, and/or new ones may be introduced in the Golgi apparatus. After these structural processings, the mature proteins may be secreted into the extracellular space (soluble proteins), or sorted into other cellular organelles, such as the plasma membrane^29,30^. Thus, it should be taken into account that a polypeptide that enters the secretory pathway might encounter a variety of structural changes as a result of misfolding and/or decoration with PTMs. These structural modifications may dramatically alter the ligand recognition and signal transduction capabilities of the protein.

To address this problem during the construction of artificial receptors, we employed a systematic approach to restore synthetic receptors’ activity in the cytoplasm, ER, and on the plasma membrane, for orthogonal signaling control. Here, we started with topology adjustment of the synthetic receptors in the cytoplasm in order to exclude the involvement of PTMs in the secretory pathway. This spatial engineering focuses on the functional evaluation of different topologies of the receptor parts in a combinatory manner. Evaluating receptors’ activities in the cytoplasm is a critical step before starting with compartment-oriented engineering, as a nonfunctional receptor in the cytoplasm is most likely to be inactive also inside the secretory organelles. Following their successful construction in the cytoplasm, active receptors were then targeted into the secretory pathway. We found that following their translocation into the secretory organelles, most receptors lost their inducibility, but we could rescue the expected activity of all these receptors by blocking functionally harmful PTMs through systematic site-directed mutagenesis of PTM-susceptible residues. Here, we chose three different small molecules as soluble ligands; rapamycin (RAPA), abscisic acid (ABA), and gibberellin (GA), collectively referred to as RAG. The ligand detection part of all these synthetic receptors consists of one of the protein domain pairs FRKB/FPB, ABI/PYL1, and GAI/GID1 that dimerize upon the addition of RAPA, ABA, or GA, respectively^31–35^. The site-specific split form of tobacco etch virus protease (TEVp) was used as a signal transducer domain in cytoplasmic and the ER-localized receptors^36,37^. In the cell membrane, we used the Notch1 receptor core. We think that these signaling moieties will be valuable tools for fine-tuning orthogonal cell signaling in mammalian cells, and they can be re-purposed during the engineering of protein architectures within the secretory pathway for cell-based medicines, diagnostics, and basic science applications.

## Results

### Engineering a membrane-docked, dormant signaling effector activated by topologically adjusted cytoplasmic receptors

**Fig. 1:**
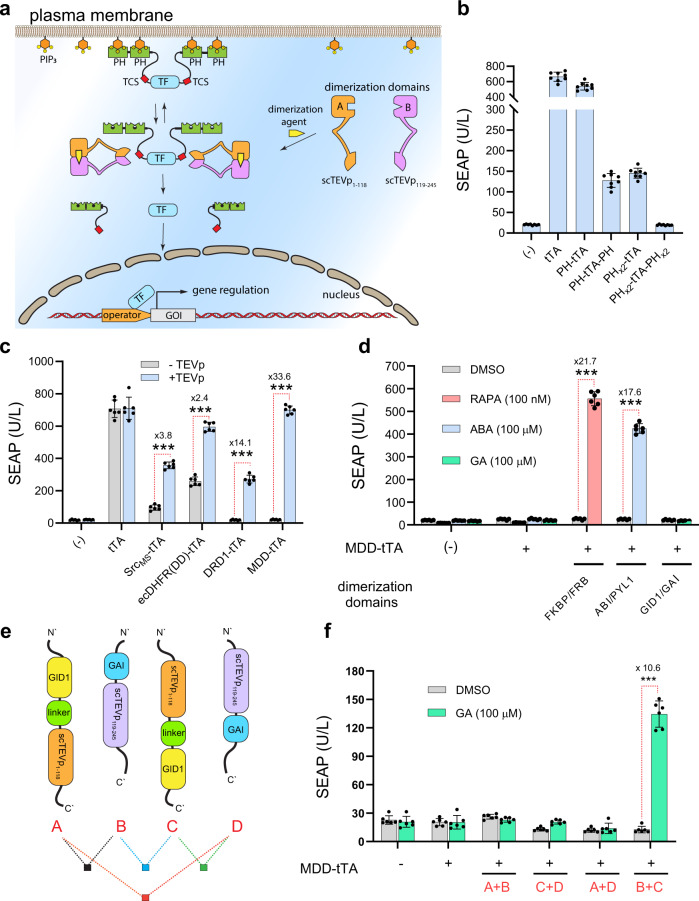
Engineering RAG-inducible intracellular receptors using a membrane-docked, dormant transcription factor for orthogonal cell signaling control. **a** Schematic illustration showing the molecular mechanism of chemically regulated intracellular receptors using membrane-docked, dormant transcription factor (MDD-TF). **b** Basal activity analysis of different tTA-PH hybrids. SEAP levels in culture supernatants of HEK-293 cells transfected with 5 ng of pMMH171 (P_hPGK_-tTA-pA), pMMH172 (P_hPGK_-PH-tTA-pA), pMMH173 (P_hPGK_-PH-tTA-PH-pA), pMMH174 (P_hPGK_-PH_x2_-tTA-pA), or pMMH125a (P_hPGK_-PH_x2_-tTA-PH_x2_-pA) plasmids. All samples were transfected with 20 ng of reporter plasmid pTS1017, which encodes tetO_7_-driven SEAP (P_hCMV*−1_-SEAP-pA_bGH_; P_hCMV*−1_, O_tetO7_-P_hCMVmin_). Data are presented as mean ± SD of *n* = 8 biologically independent samples. **c** SEAP levels after 24 h in culture supernatants of HEK-293 cells transfected with 5 ng of pMMH201 (P_hPGK_-Src_MS_-TCS-tTA-pA_bGH_), pMMH202 (P_hPGK_-ecDHFR(DD)-TCS-tTA-pA_bGH_), pMMH203 (P_hPGK_-DRD1-TCS-tTA-pA_bGH_), or pMMH125b which encode MDD-tTA (P_hPGK_-PH_x2_-TCS-tTA-TCS-PH_x2_-pA) in combination with pcDNA3.1(+) or cytoplasmic intact TEVp pTS2405 (P_hCMV_-TEVp-pA). Data are shown as mean ± SD of *n* = 6 biologically independent samples. ***P < 0.0001 was calculated using two-tailed, unpaired Student’s *t*-test. **d** SEAP levels in culture supernatants of HEK-293 cells transfected with 5 ng of pMMH125b in addition to 1 ng of pMMH139 (P_hCMV_-FKBP-(GGGGS)_2_-scTEVp_1–118_-pA_bGH_) with pMMH140 (P_hCMV_-FRB-scTEVp_119–245_-pA_bGH_), pPW20 (P_hCMV_-ABI-(GGGGS)_2_-scTEVp_1–118_-pA_bGH_) with pPW21 (P_hCMV_-PYL1-scTEVp_119–245_-pA_bGH_), or pPW22 (P_hCMV_-GID1-(GGGGS)_2_-scTEVp_1–118_- pA_bGH_) with pPW23 (P_hCMV_-GAI-scTEVp_119–245_-pA_bGH_). Cells were treated with DMSO, RAPA (100 nM), ABA (100 µM), or GA (100 µM) for 24 h. Data are presented as mean ± SD of *n* = 6 biologically independent samples. ***P < 0.0001 was calculated using two-tailed, unpaired Student’s *t*-test. **e** Illustrative depiction of different spatial topologies of GID1/GAI chimeras that are linked to csTEVp parts, annotated A–D. **f** HEK-293 cells were transfected with A–D constructs in different transfection combinations using 5 ng of pMMH125b and 1 ng of pPW22 (P_hCMV_-GID1-(GGGGS)_2_-scTEVp_1–118_-pA_bGH_), pPW23 (P_hCMV_-GAI-scTEVp_119–245_-pA_bGH_), pPW24 (P_hCMV_-scTEVp_1–118_-(GGGGS)_2_-GID1-pA_bGH_), pPW25 (P_hCMV_-scTEVp_119–245_-GAI-pA_bGH_). SEAP levels were measured 24 f following GA (100 µM) treatment. Data are presented as mean ± SD of *n* = 6 biologically independent samples. ***P < 0.0001 was calculated using two-tailed, unpaired Student’s *t*-test. Source data are provided as a Source Data file.

For reliable activity assessment of synthetic receptors in the cytoplasm, and to engineer an efficient protease-activated expression system, we first built an orthogonal signaling effector. This modular mediator is composed of a transcription factor (TF) flanked with two successive protease-cleavable pleckstrin homology (PH) domains. PH domains are evolutionarily conserved β-sandwich protein constructs found in various proteins in mammalians. They possess a strong and highly specific binding affinity for cell membrane-anchored phosphoinositides, and play a key role in targeting different proteins to the inner part of the cell membrane^38–41^. We hypothesized that decorating a TF with PH domains would cause it to be docked in the plasma membrane and would thus diminish its availability in the nucleus. In addition to their membrane-directing features, multiple PH structures generate a remarkable steric hindrance, which cooperatively reduces the basal TF activity. Thus, proteolytic cleavage of the PH domains should liberate an active form of TF that can initiate gene regulation of protein of interest (POI) (Fig. 1a). For proof of concept, we initially used tTA (tetracycline-controlled transactivator) as a transcription factor, and SEAP (human placental secreted alkaline phosphatase) as a sensitive and quantitative reporter gene.

Since the basal activity of most inducible gene switches dramatically influences their performance and robustness, we first focused on the engineering of PH-tTA chimeras with low basal activity. To do this, we systematically investigated the basal performance of a series of PH-tTA fusions constructed in various fashions (Fig. 1b). These constructs were co-transfected with a fixed amount of tTA-regulated reporter plasmid, tetO_7_-SEAP, in HEK-293 cells. As can be seen from the SEAP levels in the supernatants, the addition of one PH domain to tTA diminished the basal activity as expected; however, the decrease was not sufficient. Therefore, we systematically incorporated multiple PH domains in different orientations. We found that the PH_x2_-tTA-PH_x2_ hybrid exhibited the lowest basal leakiness, with a SEAP output comparable to that of reporter gene-expressing cells. Since the basal activity was negligible, we focused on the PH_x2_-tTA-PH_x2_ scaffold for further engineering. To explore whether the removal of PH domains enables tTA-mediated transcription, two TEVp cleavage sites (TCS) ENLYFQ^▼^S were introduced between tTA and the PH_x2_ adducts to yield a membrane-docked, dormant tTA (MDD-tTA), PH_x2_-TCS-tTA-TCS-PH_x2_. The cotransfection of MDD-tTA along with an intact cytoplasm-located TEVp expression vector led to activation, and a significant increase (33.6-fold change) in SEAP levels was observed (Fig. 1c). The performance of MDD-tTA was also compared to other strategies frequently employed in protease-activated gene circuits. The performance of tTA fusions with a TEVp-cleavable membrane-directing Src myristylation signal (MS), *Escherichia coli* dihydrofolate reductase destabilizing domain ecDHFR(DD), and dopamine receptor D_1_ (DRD1) transmembrane domain were evaluated^42,43^. As can be inferred from the SEAP levels in Fig. 1c, introducing Src_MS_ and ecDHFR(DD) resulted in leaky systems and the fold changes are around 3–4 fold upon TEVp expression. Fusing of DRD1, however, could significantly reduce the basal expression, but the fold change was not high as that of MDDA-tTA (14.1 vs 33.6), which exhibits activation output comparable to that of unmodified tTA.

In order to engineer chemically inducible systems, we utilized FKBP/FRB, PYL1/ABI, and GAI/GID1 protein-dimerizing domains that become juxtaposed upon the addition of RAPA, ABA, or GA, respectively. Each of the dimerization domains was fused to one part of the split cytoplasmic TEVp (scTEVp), affording scTEVp_(1–118)_ and scTEVp_(119–245)_. The performance of these constructs was evaluated in HEK-293 cells in the absence or presence of the relevant DA (dimerization agent). From the SEAP levels in the supernatants (Fig. 1d), we can conclude that FKBP/FRB, PYL1/ABI fusions liberate tTA when stimulated by RAPA or ABA, though the GAI/GID1 split system proved unresponsive to GA treatment. While the dimerization ability of GAI/GID1 in the presence of GA is well validated by many studies, they have rarely been used in the reconstitution of split protease systems^33,44,45^. We considered that in addition to GAI/GID1 dimerization, a specific orientation of scTEVp parts may also be required for functional reconstitution of this split system. To test this idea, we constructed different structural topologies of GAI/GID1 that are N’ or C’ terminally fused to scTEVp_(1–118)_/scTEVp_(119–245)_, annotated A-D, to cover all folding possibilities (Fig. 1e). The performance of these constructs was monitored in HEK-293 cells in a combinatory manner. We found that only one mode of assembly, B+C, could successfully restore scTEVp proteolytic activity in response to GA treatment (Fig. 1f).

### Engineering ER-dwelling receptors by blocking activity-destructive disulfide bridge formation

**Fig. 2:**
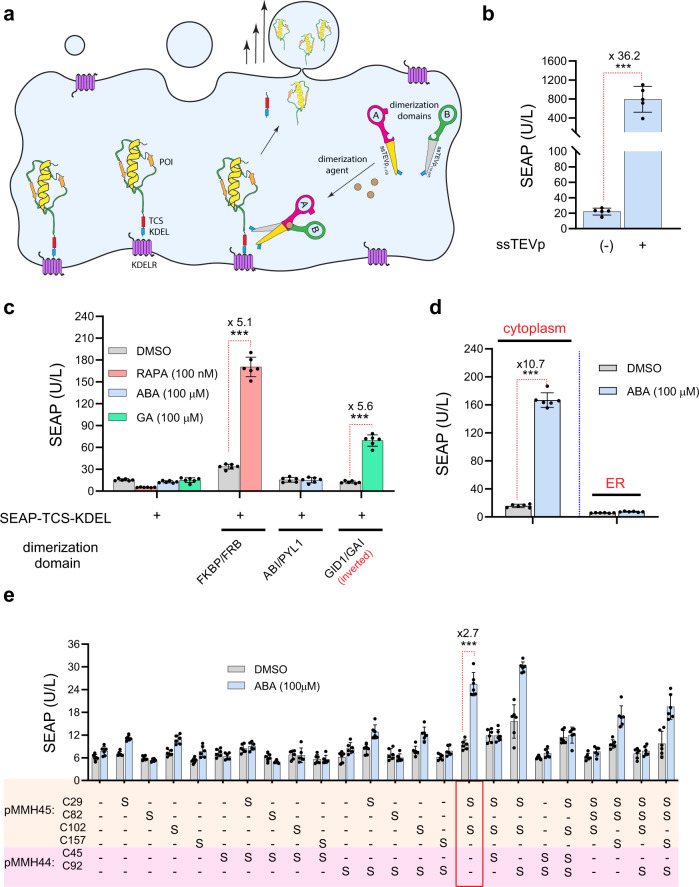
Design of RAG-inducible modules for fast secretion control of ER-accumulated glycoproteins with avoidance of disulfide bridge formation in CID domains. **a** Schematic illustration of the molecular components that comprise the endoplasmic reticulum RAG-inducible system. **b** SEAP levels after 24 h in culture supernatants of HEK-293 cells transfected with 5 ng of pMMH51 and pMMH10 plasmids, which encode intact secTEVp-KDEL (P_hCMV_-ssTEVp-KDEL-pA) and SEAP-TCS-KDEL (P_hCMV_-SEAP-TCS-KDEL-pA) separately or in combination. Data are presented as mean ± SD of *n* = 5 biologically independent samples. ***P < 0.0001 was calculated using two-tailed, unpaired Student’s *t*-test. **c** SEAP levels in culture supernatants of HEK-293 cells transfected with pMMH10 (5 ng) with pMMH26 (P_hCMV_-SS-FKBP-(GGGGS)_2_-ssTEVp_1–118_-KDEL-pA) and pMMH27 (P_hCMV_-SS-FRB-ssTEVp_119–245_-KDEL-pA), pMMH44 (P_hCMV_- SS-PYL1-ssTEVp_119–245_-KDEL-pA_bGH_) and pMMH45 (P_hCMV_- SS-ABI-(GGGGS)_2_-ssTEVp_1–118_-KDEL-pA_bGH_), pMMH43 (P_hCMV_-SS-GAI-ssTEVp_119–245_-KDEL -pA_bGH_) and pPW26 (P_hCMV_-SS-ssTEVp_1–118_-(GGGGS)_2_-GID1-KDEL-pA_bGH_) (5 ng each). Cells were treated with DMSO, RAPA (100 nM), ABA (100 µM), or GA (100 µM). Data are presented as mean ± SD of *n* = 6 biologically independent samples. ***P < 0.0001 was calculated using two-tailed, unpaired Student’s *t*-test. **d** Left: SEAP levels in culture supernatants of HEK-293 cells transfected with pMMH125b, which encodes MDD-tTA, along with 1 ng of the cytoplasmic pPW20 (P_hCMV_-ABI-(GGGGS)_2_-ssTEVp_1–118_-KDEL-pA_bGH_) and pPW21 (P_hCMV_-PYL1-ssTEVp_119–245_-KDEL-pA_bGH_). Right: SEAP levels in the supernatants of cells transfected with the ER-resident counterparts, e.g., pMMH44 (P_hCMV_- SS-PYL1-ssTEVp_119–245_-KDEL-pA_bGH_) and pMMH45 (P_hCMV_-SS-ABI-(GGGGS)_2_-ssTEVp_1–118_-KDEL-pA_bGH_), in combination with 5 ng of the reporter gene pMMH10, which encodes SEAP-TCS-KDEL (P_hCMV_-SEAP-TCS-KDEL-pA). Transfected cells were treated either with DMSO or ABA (100 µM). Data are presented as mean ± SD of *n* = 6 biologically independent samples. ***P < 0.0001 was calculated using two-tailed, unpaired Student’s *t*-test. **e** Site-directed mutagenesis of ER-localized ABI and PYL1 dimerization domains fused with ssTEVp_1–118_ and ssTEVp_119–245_, respectively. SEAP levels in culture supernatants of cells transfected with 5 ng pMMH10 in addition to 5 ng of different S to C mutants of pMMH44 (P_hCMV_-SS-PYL1-ssTEVp_119–245_-KDEL-pA_bGH_) and pMMH45 (P_hCMV_-SS-ABI-(GGGGS)_2_-ssTEVp_1–118_-KDEL-pA_bGH_). Data are presented as mean ± SD of *n* = 6 biologically independent samples. ***P < 0.0001 was calculated using two-tailed, unpaired Student’s *t*-test. Source data are provided as a Source Data file.

We previously engineered the endoplasmic-reticulum-localized split secTEVp-based rapamycin-actuated protein-induction device (RAPID) as a fast-releasing system for glycoproteins, using rapamycin (RAPA) as signal initiator^46^. Other recent studies have also employed similar strategies for engineering gene circuits^47,48^. In this system, the protein of interest (POI) is C’ terminally tagged with KDEL, a retention signal that prevents protein emigration from the ER due to its constitutive interaction with ER-dwelling KDEL receptors (Fig. 2a). Here, the KDEL signal is designed to be proteolytically cleaved from the POI by secTEVp (by introducing TCS between the POI and KDEL), allowing secretion of the POI into the extracellular space within a few minutes following RAPA addition. The cotransfection of SEAP-TCS-KDEL with intact secTEVp significantly increased the SEAP level in the supernatant, compared to cells that express SEAP-TCS-KDEL alone (Fig. 2b). To engineer chemically inducible systems, protein-dimerizing domains FKBP/FRB, PYL1/ABI, and GAI were fused N’ terminally with a secretion signal in order to target the receptors into the secretory pathway. Exceptionally, GID1 was inverted relative to other constructs, as we noted before (Fig. 1e, f). Split secTEVp domains, ssTEVp_1–118_ and ssTEVp_119–245_, were introduced with the same topologies that were functional in the cytoplasm (Fig. 1d, f). In addition, these split constructs were engineered with secTEVp non-cleavable KDEL tags in order to prevent their auto-proteolysis and consequent secretion. The inducibility of these constructs was evaluated in HEK-293 cells, using SEAP-TCS-KDEL as a reporter gene. The RAPA-regulated system was inducible as we previously reported, and only the topologically engineered version of GID1/GAI (the inverted form) responded to GA as shown in Fig. 1f. However, the ABA-regulated system did not respond to inducer addition (Fig. 2c).

Interestingly, when ABA-regulated receptors were re-targeted from the ER to the cytoplasm (by removing the secretion signal and retaining TCS-KDEL), they regained their proteolytic activity when the cytoplasmic MDD-tTA (see Fig. 1) was used instead (Fig. 2d). This result implies that ABI/PYL1-ssTEVp receptors undergo ER-mediated PTMs that alter their physicochemical features and impair their dimerization ability in the presence of ABA. The formation of disulfide bonds between distantly located cysteine residues plays a pivotal role in shaping protein spatial structures in the secretory pathway, and the ER is the major cellular compartment where S-S bridges are stably formed by ER-resident enzymes, the protein disulfide isomerases (PDIs)^49^. In order to rescue these ER-retained receptors from functionally detrimental PTMs, we decided to block the formation of specific S-S bridges in the ABI /PYL1 dimerization domains by site-directed mutagenesis of cysteine residues. For this purpose, we performed a systematic mutagenesis screen, replacing cysteine with serine residues (Fig. 2e). The reason why we selected serine is its structural similarity to cysteine (the only difference is the oxygen atom instead of sulfur in cysteine), and its inability to form inter/intramolecular bridges. As can be seen from the SEAP levels in the supernatants of the cells transfected with the different mutants, singly mutated analogs could not rescue these constructs, so we next examined systematic double and triple mutagenesis. Fortunately, we found that ABI double-mutated at C29 and C102 could partially rescue the dimerization capability of PYL1/ABI in the presence of ABA when expressed in the ER.

### Engineering an efficient ABA-inducible fast-release system through combinatory mutagenesis of N-linked glycosylation sites and disulfide bond-forming residues

**Fig. 3:**
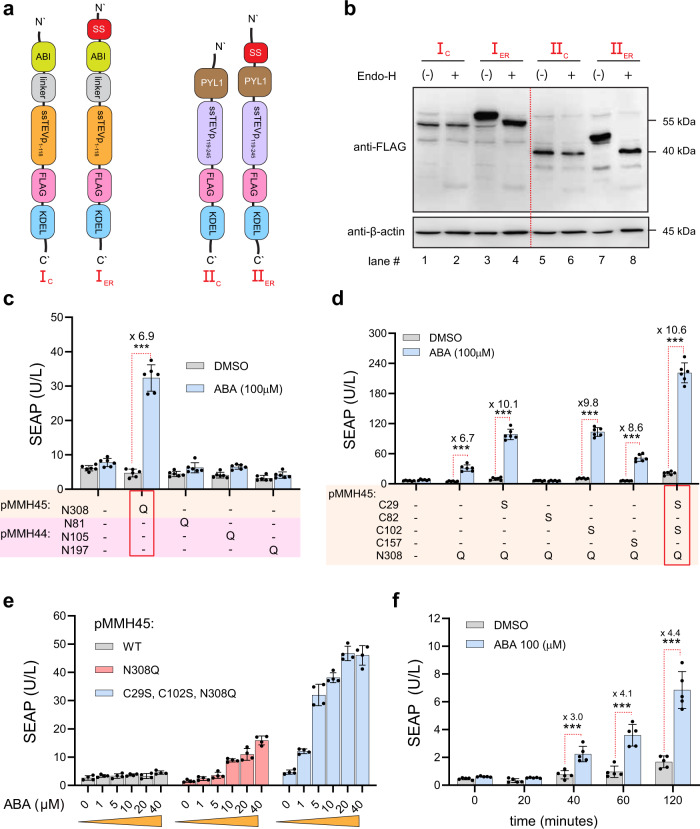
Engineering an efficient ER-localized ABA-inducible fast-release system through combinatory mutagenesis of C and N residues in CIDs to circumvent functionally detrimental PTMs. **a** Structure depiction of the cytoplasmic and the ER-localized versions of ABI-ssTEVp_1–118_ and PYL1-ssTEVp_119–245_, annotated as I_c_ (pPW32), I_ER_ (pPW34), II_c_ (pPW33), and II_ER_ (pPW35). A secretion signal (SS) was introduced into I_ER_ and II_ER_ fusions. All constructs were tagged with FLAG for further analysis. **b** Immunoblotting against FLAG tag of HEK-293 cells transfected separately with the I_c_, I_ER_, II_c_, or II_ER_ following endo-H treatment of the total lysates. Actin was used as a loading control. **c** Site-directed mutagenesis of ER-localized ABI-ssTEVp_1–118_ and PYL1-ssTEVp_119–245_ fusion proteins. SEAP levels in culture supernatants of HEK-293 cells transfected with 5 ng pMMH10, which encodes SEAP-TCS-KDEL (P_hCMV_-SEAP-TCS-KDEL-pA), in addition to 5 ng of different N to Q mutants of pMMH44 (P_hCMV_-SS-PYL1-ssTEVp_119–245_-KDEL-pA_bGH_) and pMMH45 (P_hCMV_-SS-ABI-(GGGGS)_2_-ssTEVp_1–118_-KDEL-pA_bGH_). Data are presented as mean ± SD of *n* = 6 biologically independent samples. ***P < 0.0001 was calculated using two-tailed, unpaired Student’s *t*-test. **d** Combinatory site-directed mutagenesis of ER-localized ABI-ssTEVp_1–118_, pMMH45. SEAP levels in culture supernatants of cells transfected with 5 ng pMMH10 in addition to 5 ng of different N to Q and/or C to S mutants of pMMH45 with non-mutagenized pMMH44_WT_. Data are presented as mean ± SD of *n* = 6 biologically independent samples. ***P < 0.0001 was calculated using two-tailed, unpaired Student’s *t*-test. **e** Dose-response analysis of pMMH45_WT_ pMMH45_N308Q_, and pMMH45_C29S, C102, N308Q_ mutants. Data are presented as mean ± SD of *n* = 4 biologically independent samples. **f** SEAP levels in culture supernatants of HEK-293 cells transfected with pMMH10, pMMH44_WT_, and pMMH45_C29S, C102, N308Q_ (5 ng each). At 24 h after transfection, the culture medium was replaced with 30 µl of fresh medium containing either DMSO or ABA (100 µM) and 20 µl aliquots were collected for analysis at different time points. Data are presented as mean ± SD of *n* = 5 biologically independent samples. ***P < 0.0001 was calculated using two-tailed, unpaired Student’s *t*-test. Source data are provided as a Source Data file.

To enhance the activity of ABI/PYL1-based receptors inside the ER, we also investigated the involvement of asparagine (Asn) N-linked glycosylation, since the addition of bulky sugars to synthetic moieties may modify their structures and affect the expected activity. Biochemically, N-linked glycosylation consists of the selective attachment of glycans to the nitrogen atom of asparagine within the canonical Asn–X–Ser/Thr consensus sequence, and this common PTM occurs mainly in the ER^50^. To explore whether ABI/PYL1 undergo such PTMs, we initially constructed both ER- and cytoplasm-localized ABI/PYL1 receptors with ssTEVp_1–118_ and ssTEVp_119–245_, as depicted in Fig. 3a (annotated as I_C_, I_ER_, II_C_, II_ER_). The only difference between the cytoplasmic versions and the ER-dwelling analogs is the presence of the secretion signal (SS). All of these constructs were FLAG-tagged just before the KDEL retention signal, to enable their detection by western blotting. To monitor whether these constructs undergo N-linked glycosylation, we transfected these chimeras into HEK-293 cells and prepared the total lysate from each sample. The cleared lysates were then treated with endo-H, an endoglycosidase that cleaves asparagine-linked oligosaccharides^51^. The resulting lysates were separated by SDS-PAGE and immunoblotted against FLAG tag (Fig. 3b). Receptors inserted into the ER were endo-H sensitive, as they exhibited lower molecular weight due to the removal of covalently attached sugar (lane #3 vs #4 and lane #7 vs #8). In contrast, the cytoplasmic versions were not affected by endo-H treatment (lane #1 vs #2 and lane #5 vs #6). These results indicate that ABI/PYL1-ssTEVp constructs do undergo N-linked glycosylation.

To investigate whether these receptors could be rescued from potentially detrimental N-linked glycosylation, we conducted asparagine (N) to glutamine (Q) mutagenesis analysis of PYL1 and ABI for asparagine residues located within the Asn–X–Ser/Thr consensus motif (X is any amino acid except proline) (Fig. 3c). Indeed, asparagine 308 in ABI appears to be involved in N-linked glycosylation, because converting this amino acid to glutamine significantly improved its dimerization ability with PYL1_WT_ in the presence of ABA. To further improve the ABA-inducibility of ABI/PYL1-ssTEVp, we performed combinatory mutagenesis analysis using ABI_N308Q_ with the C29S/C102S mutations that we had already characterized (Fig. 2e). It is clear from the SEAP levels in the supernatants that triple-mutated ABI_N308Q, C29S, C102S_ is effectively induced inside the ER, showing more than 10-fold change versus nonfunctional ABI_WT_ in response to ABA (Fig. 3d). Since changing these residues may also affect the binding affinity, we next examined the binding efficiency of ABI_WT_, ABI_N308Q_ and ABI_N308Q/ C29S/C102S_ with PYL1_wt_. A dose-response analysis showed that the introduced mutations did not affect the binding affinity, and the EC_50_ value was maintained at around ~4 µM (Fig. 3e)^31^. To demonstrate a potential application of the engineered ABI_N308Q/ C29S/C102S_, a fast-release experiment was conducted in HEK-293 cells. The cells were transfected with SEAP-TCS-KDEL, ABI_N308Q/ C29S/C102S_-ssTEVp_1–118_, and PYL1_WT_-ssTEVp_119–245_. Indeed, the addition of ABA triggered the secretion of SEAP into the supernatant in less than 40 min (Fig. 3f).

### Engineering of cis-functional transmembrane ABA-regulated orthogonal chemically activated receptors (OCARs)

**Fig. 4:**
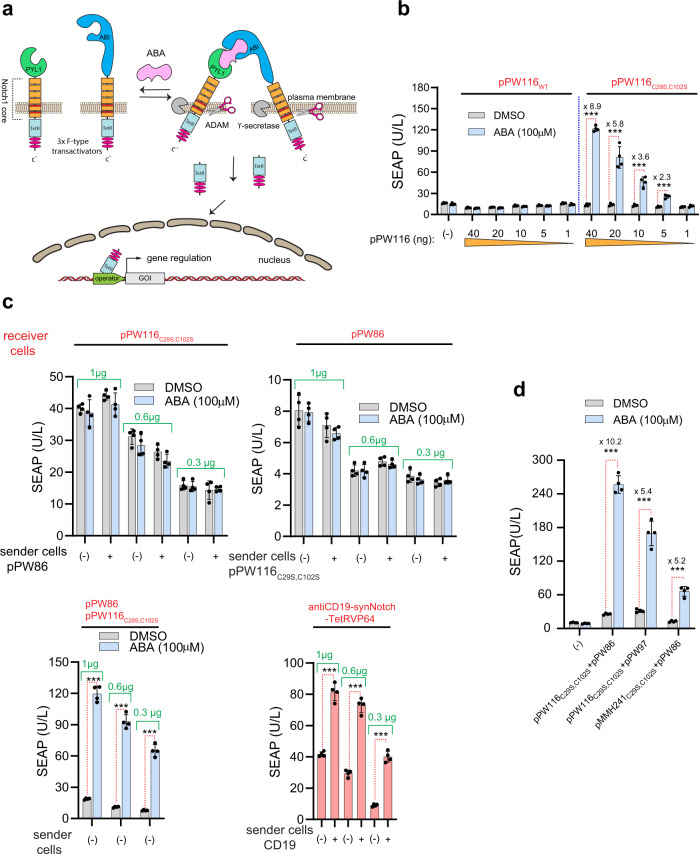
Engineering the orthogonal chemically activated receptor (OCAR) platform for selective and orthogonal cell signaling control. **a** Schematic representation of the ABA-regulated OCAR. **b** SEAP levels in the supernatants of cells transfected with 40 ng pPW86_WT_ (P_PGK_-SS-PYL1_WT_-Notch1_core_-TetR-VP64-pA) and with different amounts of either pPW116_WT_ (P_PGK_-SS-ABI_WT_-Notch1_core_-TetR-VP64-pA) or pPW116_C29S,C102S_ (P_PGK_-SS-ABI _C29S,C102S_-Notch1_core_-TetR-VP64-pA). All samples were transfected with 20 ng of reporter plasmid pTS1017, which encodes to tetO_7_-driven SEAP (O_tetO7_-P_hCMVmin_ TetO_7_-SEAP). Data are presented as mean ± SD of *n* = 4 biologically independent samples. ***P < 0.0001 was calculated using two-tailed, unpaired Student’s *t*-test. **c** SEAP levels in the supernatants of co-cultured HEK-293 cells. Receiver cells were transfected with different amounts of pPW116_C29S,C102S_ and pPW86_WT_ either separately or in combination. Similar transfection procedure was performed using anti-CD19-synNotch-TetR-VP64. All receiver cells were co-transfected with the reporter plasmid pTS1017 (see methods). Sender cells were separately transfected either with pPW116_C29S,C102S_, pPW86_WT_, or with CD19 expression vector INS-2A-luciferase-2A-CD19-GFP. pcDNA3.1 (+) transfected HEK-293 sender cells are indicated as (–). ABA and DMSO were immediately added to the mixed cells, and SEAP measurements were performed 24 h following the co-culturing. Data are presented as mean ± SD of *n* = 4 biologically independent samples. ***P < 0.0001 was calculated using two-tailed, unpaired Student’s *t*-test. **d** SEAP levels in the supernatants of cells transfected with 40 ng pPW86_WT_, pPW116_C29S,C102S_, pPW97 (P_hPGK_-SS-PYL1-Notch1_core_-pA_bGH_), and pMMH241_C29S,C102S_ (P_hPGK_-SS-ABI_C29S,C102S_-Notch1_core_-pA_bGH_). All samples were transfected with 20 ng of reporter plasmid pTS1017. Data are presented as mean ± SD of *n* = 4 biologically independent samples. ***P < 0.0001 was calculated using two-tailed, unpaired Student’s *t*-test. Source data are provided as a Source Data file.

The main disadvantage of integrating a synthetic gene switch into a cell’s endogenous pathways is the poor predictability of the activation pattern, because many endogenous signaling cascades are highly interconnected, and are stimulated by multiple signal inputs. Thus, orthogonal pathways are needed to ensure high selectivity and operational reliability, especially for in vivo applications. We therefore engineered an orthogonal ABA-activated receptor in the plasma membrane. For this system, which we call orthogonal chemically activated receptor (OCAR), we utilized the Notch1 receptor core transmembrane domain (Notch1_core_)^7^. This core architecture was N’ terminally connected with ABA-sensitive protein-dimerizing domains ABI and PYL1. At the C’ terminus, however, these receptors were fused to a cleavable tTA for gene regulation. Mechanistically, upon the addition of the dimerization-inducing agent (DA), the OCAR parts bind to each other and undergo conformational change. These changes in the receptor parts stimulate their cleavage by endogenous membrane-anchored proteases (such as ADAM and γ-secretase) (Fig. 4a). Once liberated, the tTA translocates to the cell nucleus, and controls gene expression.

In this study, we used TetR as a DBD (DNA binding domain) and VP64 as a transcriptional transactivator. SEAP under tetO_7_ regulation was used as the reporter. Cotransfection of HEK-293 cells with pPW86_WT_ (P_PGK_-SS-PYL1_WT_-Notch1_core_-TetR-VP64-pA) together with different amounts of pPW116_WT_ (P_PGK_-SS-ABI_WT_-Notch1_core_-TetR-VP64-pA) did not result in any marked change in SEAP levels when ABA was added (Fig. 4b, left half). Analysis of the primary structure of Notch1_core_ revealed that this scaffold contains an exceptionally large number of cysteine residues (21 residues), which comprise more than 15% of the total amino acids. Thus, functionally detrimental disulfide bond formation between Notch1_core_ and the dimerization domains was considered likely to be responsible for the non-inducibility of OCAR parts. To validate this hypothesis, we systematically replaced cysteine residues with serine residues. First, we showed that C29S and C102S mutations of ABI rescued ABI/PYL1 dimerization of the ER receptor (Fig. 2e). Then, to validate these mutations in the OCAR system, we designed ABI_C29S,C102S_-Notch1_core_-TetR-VP64 (pPW116_C29S,C102S_). We found that this synthetic OCAR receptor was indeed functional in HEK-293 cells after cotransfection with its heterodimerizing companion receptor (pPW86_WT_, SS-PYL1-Notch1_core_-TetR-VP64), in contrast to its wild-type version (pPW116_WT_) (Fig. 4b). To elucidate its activation mode, we performed co-culturing experiments to examine whether ABA-regulated OCAR works in cis (on the same cell) or in trans upon inducer addition. To explore this, two cell populations were separately transfected with one of the receptor parts, and subsequently, they were co-cultured in absence/presence of ABA. We also co-transfect both receptor parts in the same population. As a positive control, we use trans-acting anti-CD19 SynNotch^7^. In ABA-induced OCAR, co-culturing of the two cell populations that express one of the receptor parts did not lead to any marked increase in SEAP levels when ABA was added (Fig. 4c, top left and right), in contrast to cells that simultaneously transfected with both parts of the receptor (Fig. 4c, bottom left). Using the same experimental settings, we found that anti-CD19 synNotch-expressing cells are stimulated when co-cultured with CD19-overexpressing cells (Fig. 4c, bottom right), suggesting that ABA-inducible OCAR functions in a cis fashion. To investigate which intracellular part of ABA-regulated OCAR is cleaved following the inducer addition, we tested the activity of pPW86_WT_ and pPW116_C29S,C102S_ with their TetR-VP64-lacking counterparts pPW97 (P_hPGK_-SS-PYL1-Notch1_core_-pA_bGH_) and pMMH241_C29S,C102S_ (P_hPGK_-SS-ABI_C29S,C102S_-Notch1_core_-pA_bGH_), respectively (Fig. 4d). From the SEAP levels in the supernatants, we can conclude that both intracellular parts of the receptor (TetR-VP64) are cleaved in the presence of ABA and both contribute to the overall SEAP signal.

### Engineering RAPA- and GA-inducible OCARs through systematic mutagenesis of cysteine residues in the receptor-dimerizing domains

To expand the OCAR platform for other dimerization agents (DA), and to show that cysteine mutagenesis can rescue the receptors’ activity, we engineered RAPA- and GA-inducible OCAR systems.

**Fig. 5:**
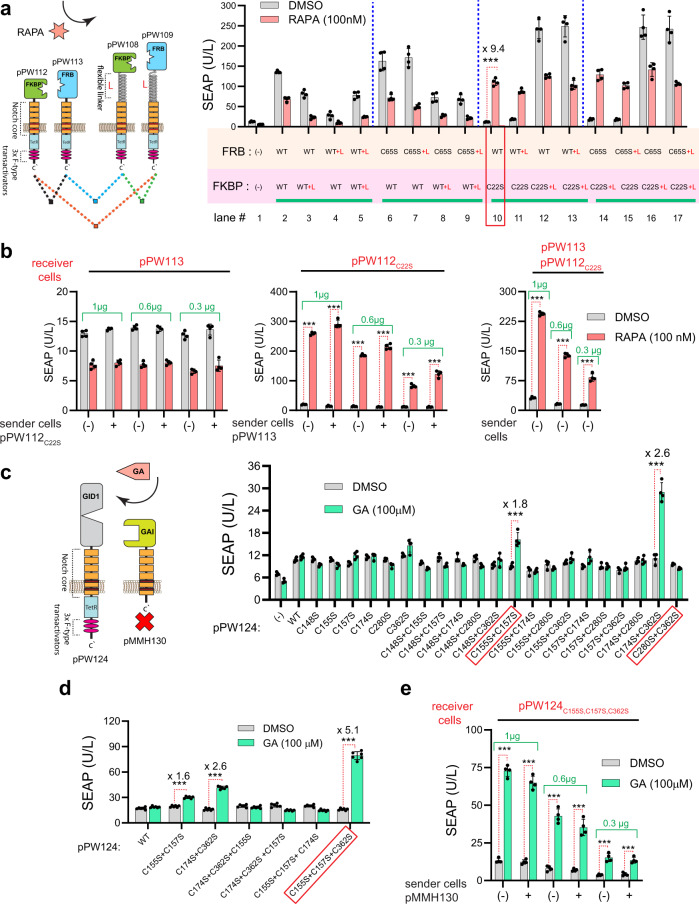
Rational engineering of RAPA- and GA-regulated OCARs by C to S site-directed mutagenesis in CID domains. **a** Left: structural architectures of the initial, non-mutagenized constructs pPW108 (P_PGK_-SS-FKBP-(GGGS)_x2_-Notch1_core_-TetR-VP64-pA), pPW109 (P_PGK_-SS-FRB-(GGGS)_x2_-Notch1_core_-TetR-VP64-pA), pPW112, and pPW113. “L” symbolizes a flexible linker. Right: SEAP levels in supernatants of HEK-293 cells transfected with pPW108, pPW109, pPW112, and pPW113 using either WT and/or different C to S mutants (40 ng each). Data are presented as mean ± SD of *n* = 4 biologically independent samples. ***P < 0.0001 was calculated using two-tailed, unpaired Student’s *t*-test. **b** SEAP levels in the supernatants of co-cultured cells. Receiver cells were transfected with different amounts of pPW113 and pPW112_C22S_ either separately or in combination (see methods). Sender cells were separately transfected either with pPW112_C22S_ or pPW113. RAPA and DMSO were immediately added to the mixed cells, and SEAP measurements were performed 24 h following the co-culturing. Data are presented as mean ± SD of *n* = 4 biologically independent samples. ***P < 0.0001 was calculated using two-tailed, unpaired Student’s *t*-test. **c** Systematic C to S mutagenesis of pPW124 (P_PGK_-SS-GID1-Notch1_core_-TetR-VP64-pA). Cells were transfected with pMMH130 (P_PGK_-SS-GAI_WT_-Notch1_core_-pA) and different C to S mutants of pPW124 (40 ng each). SEAP levels were measured at 24 h after GA (100 µM) treatment. Data are presented as mean ± SD of *n* = 4 biologically independent samples. ***P < 0.0001 was calculated using two-tailed, unpaired Student’s *t*-test. **d** Combinatory C to S mutagenesis analysis of pPW124 and SEAP was measured as described in (**c**). Data are presented as mean ± SD of *n* = 6 biologically independent samples. ***P < 0.0001 was calculated using two-tailed, unpaired Student’s *t*-test. **e** SEAP levels in the supernatants of co-cultured cells. Sender cells were transfected with pMMH130 and receiver cells were transfected with different amounts of pPW124_C155S, C157S, C362S_. GA was used as the inducer. Data are presented as mean ± SD of *n* = 4 biologically independent samples. ***P < 0.0001 was calculated using two-tailed, unpaired Student’s *t*-test. Source data are provided as a Source Data file.

Here, we fused FKBP/FRP dimerization domains to Notch1_core_-TetR-VP64, as depicted in Fig. 5a (left). To avoid increasing the rigidity of the protein, which may hamper the ability of FKBP/FRP to dimerize upon RAPA addition, we introduced a flexible linker (GGGGS)_x2_ between these domains and Notch1_core_. Activity screening of a series of constructs was performed to evaluate the effect of linker presence and C to S mutagenesis of FKBP/FRP. Transfection of HEK-293 cells with these receptors in different combinatory patterns demonstrated that C22S mutation in FKBP restored OCAR functionality (lane #10), resulting in 9.4-fold induction (Fig. 5a, right). The presence of a flexible linker in the FKBP_C22S_ mutant seems to have a negative impact on its activity, and significantly increases the basal expression (lane #12). Increased leakiness was also observed when a (GGGGS)_x2_ linker was introduced into FRB, even though inducibility was preserved (lane #10 vs #11). RAPA treatment decreased the basal expression of nonfunctional receptors, and this can be also seen in cells that express reporter gene alone. This decrease in signal is due to RAPA inhibition of the mTOR pathway, which diminishes global protein synthesis (see Fig. 5a, lanes #1–5)^52–54^. In active receptors, however, the signaling output of OCAR exceeds the RAPA-mediator protein attenuation, thereby overriding the unspecific rapamycin-mediated decrease. This was confirmed by employing the rapalog FK506, which activated OCAR without any unspecific decrease (Supplementary Fig. S2).

To evaluate whether RAPA-regulated OCAR also functions in cis, as does the ABA-induced variant (Fig. 4c), we performed the same type of co-culture experiments combining sender cells encoding SS-FRB-Notch1_core_-TetR-VP64 (pPW113) and receiver cells encoding SS-FKBPC22S-Notch1_core_-TetR-VP64 (pPW112C22S). Surprisingly, SS-FKBPC22S-Notch1_core_-TetR-VP64 alone was sufficient for RAPA-triggered activation (Fig. 5b, middle). To engineer GA-inducible OCAR, we first cloned pPW124_WT_ and pPW125_WT_ as depicted in Supplementary Fig. S1a (left), and then conducted systematic mono and di mutagenesis screening of GID1 (GAI does not contain any cysteine residue). Unfortunately, neither mono nor di-mutated GID1 could rescue GID1/GAI inducibility (Supplementary Fig. S1a, right). However, unexpected behavior was observed in this C to S mutagenesis screen in GID1. All the mutants (and the WT) showed remarkably high basal expression levels as inferred from the SEAP levels of reporter-expressing cells, denoted as (–), compared to the other samples (see red arrow). To determine which construct is responsible for this leakiness, we separately transfected pPW124_WT_ and pPW125_WT_ into HEK-293 cells (Supplementary Fig. S1b). It turned out that the transfection of pPW125_WT_ alone leads to high leakiness, which could have masked the activities of some mutants that we previously generated. To solve this problem, we removed the intracellular part of pPW125_WT_ (TetR-VP64 transactivator) to produce pMHH130 as a “silent” dimerization anchor (Fig. 5c, left), and repeated the same screen using pMHH130 and pPW124 mutants instead (Fig. 5c, right). Here, the signal intensity was significantly reduced and it can be seen that the double mutations C155S, C157S and C174S, C362S in GID1 partially restore the dimerization ability of OCAR receptors in the presence of GA (1.8- and 2.6-fold change, respectively). To further increase the fold change and to enhance GA-induced OCAR, we performed combinatory triple mutagenesis of cysteine hotspots C155S, C157S, C174S, and C362S (Fig. 5d). Finally, the triple-mutated GID1 _C155S, C157S, C362S_ showed significant induction by GA, with a more than 5-fold change. Co-culture experiments combining cells expressing the SS-GID1-Notch1_core_-TetR-VP64 variant (pPW124_C155S,C157S,C362S_) with cells encoding SS-GAI-Notch1_core_ (pMHH130) revealed that, as in the case of the RAPA-triggered OCAR, the SS-GID1-Notch1_core_-TetR-VP64 OCAR variant (pPW124_C155S,C157S,C362S_) was also sufficient for GA-triggered activation (Fig. 5e).

### OCAR platform possesses intrinsic off-switch control, and ABA-regulated OCAR can be used to increase synNotch activity during cell-cell communication

**Fig. 6:**
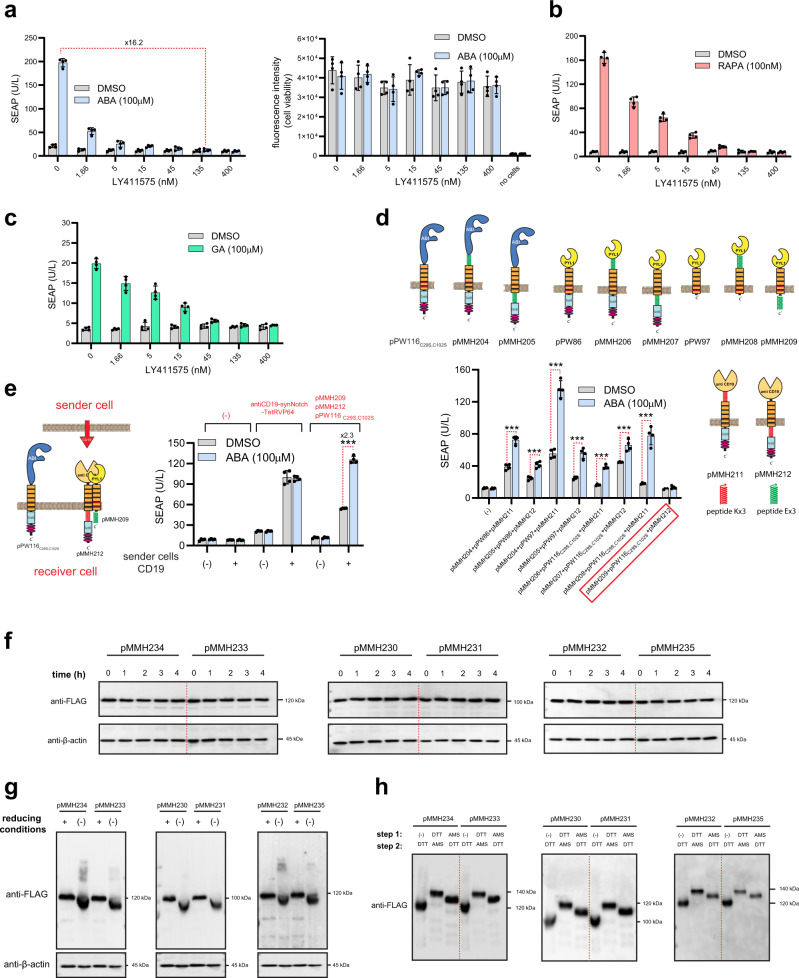
OCAR platform possesses an intrinsic off-switch control, and ABA-regulated OCAR can be used to increase synNotch activity during cell-cell communication. **a** Left: SEAP levels in the supernatants of HEK-293 cells transfected pPW116_C29S,C102S_, pPW86_WT_, and reporter plasmid pTS1017. Following the transfection, cells were treated with different concentrations of γ-secretase inhibitor LY411575 along with ABA (100 µM) or DMSO for 24 h. Right: alamarBlue cell viability assay of the previous samples. Data are presented as mean ± SD of *n* = 4 biologically independent samples. **b**, **c** The same experiment as shown in (**a**) for OCARs regulated by RAPA (**b**) and GA (**c**). For analysis of the RAPA-regulated OCAR, the cells were transfected with pPW112_C22S_. For analysis of the GA-regulated OCAR pPW124_C155S, C157S, C362S_ was transfected. Data are presented as mean ± SD of *n* = 4 biologically independent samples. **d** SEAP levels in the supernatants of cells transfected with the indicated plasmids. Data are presented as mean ± SD of *n* = 4 biologically independent samples. ***P < 0.0001 was calculated using two-tailed, unpaired Student’s *t*-test. **e** SEAP levels in the supernatants of co-cultured cells. Receiver cells were transfected either with pMMH209, pMMH212, and pPW116_C29S,C102S_, combination or with anti-CD19-synNotch-TetR-VP64. The transfected receiver cells were co-cultured with sender cells that overexpress CD19 or sender cells that were transfected with pcDNA3.1(+), in presence/absence ABA. Data are presented as mean ± SD of *n* = 4 biologically independent samples. ***P < 0.0001 was calculated using two-tailed, unpaired Student’s *t*-test. **f** Immunoblotting against FLAG and β-actin of cells separately transfected with pMMH234 (P_hPGK_-SS-ABI-Notch1_core_-TetR-VP64-2xFLAG-pA_bGH_), pMMH233 (P_hPGK_-SS-ABI_C29S_,_C102S_-Notch1_core_-TetR-VP64-2xFLAG-pA_bGH_), pMMH230 (P_hPGK_-SS-FKBP-Notch1_core_-TetR-VP64-2xFLAG-pA_bGH_), pMMH231(P_hPGK_-SS-FKBP_C22S_-Notch1_core_-TetR-VP64-2xFLAG-pA_bGH_), pMMH232(P_hPGK_-SS-GID1-Notch1_core_-TetR-VP64-2xFLAG-pA_bGH_), and pMMH235 (P_hPGK_-SS-GID1_C155S_,_C157S_,_C362S_-Notch1_core_-TetR-VP64-2xFLAG-pA_bGH_) followed by cycloheximide chase (see Methods). **g** Immunoblotting against FLAG and β-actin of cells separately transfected with the same plasmids as above. For each sample, the cleared lysate was divided equally into two parts and sample buffer with or without DTT was added. **h** Immunoblotting against FLAG following AMS shift assay of cells transfected with the same plasmids as above (see Methods). Source data are provided as a Source Data file.

Notch receptors rely on membrane-localized proteases, ADAM and γ-secretase, for their cleavage upon binding to suitable ligands presented on the sender cells^55^. Potent and selective γ-secretase inhibitors have recently been developed and some are found in various clinical trials^56–58^. To examine whether OCAR receptors can also be regulated by γ-secretase inhibitor, we treated the cells with different concentrations of LY411575 (Fig. 6a, left). Indeed, the addition of LY411575 abolished OCAR activity despite the presence of ABA. To exclude the possibility that the effect of LY411575 is due to cell toxicity, we also evaluated cell viability using alamarBlue assay (Fig. 6a, right). Similar characteristics were also observed for RAPA and GA-stimulated OCARs (Fig. 6b, c). This built-in characteristic could be utilized as safety switch to stop gene expression during cell-based therapy. Next, we investigated whether we could influence the behavior of trans-acting SynNotch using our engineered cis-acting ABA-regulated OCAR during cell-cell communication. Here, we first sequestered one part of ABA-regulated OCAR to anti-CD19 SynNotch receptor through coiled-coil interactions in order to prevent ABA-induced activation in the absence of CD19-presenting sender cells. To do this, we cloned several constructs using high-affinity coiled-coil interacting peptides, Ex3 and Kx3, as depicted in Fig. 6d^59,60^. The effect of cotransfection of different combinations of these receptors was analyzed in the presence/absence of ABA (Fig. 6d). Interestingly, the cotransfection of pMMH209, pMMH212, and pPW116_C29S, C102S_ almost abrogated the response to ABA, suggesting an efficient intermolecular interaction between pMMH209 and pMMH212. To explore whether the co-culture of CD19-overexpressing cells (sender cells) can restore ABA-responsiveness and thus enhance SynNotch signaling by liberating pMMH212 from the receiver cells, we performed co-culturing experiments as shown in Fig. 6e. Indeed, the transfection of pMMH209, pMMH212, and pPW116_C29S, C102S_ significantly enhanced the performance of anti-CD19 SynNotch signaling when CD19-positive sender cells were co-cultured in addition to ABA. Comparative SDS-PAGE-based Western blot analysis of wild-type and mutant OCARs showed no significant changes in band size or intensity, suggesting that OCARs show similar expression levels and stability and do not form intermolecular disulfide bonds with other proteins (Fig. 6f, g). However, treatment of the OCARs with DTT and/or AMS resulted in band shifts, indicating that these receptors form intramolecular disulfide bonds (Fig. 6h).

## Discussion

In this work, we have engineered several synthetic receptors that reside in different organelles of mammalian cells by focusing on orientation adjustment of the receptor parts, and by employing rational mutagenesis to block functionally destructive PTMs in the secretory pathway. This approach enabled us to engineer various artificial receptors located in the cytoplasm, the ER, and on the cell surface, orthogonally controlled by small soluble ligands (RAPA, ABA, and GA). Here, we first engineered MDD-tTA as a robust and highly efficient signaling effector in the cytoplasm. Structurally, this signaling mediator consists of tTA, flanked with multiple TEVp-cleavable PH domains, which serve as a bulky steric shield and promote transfer to the membrane, synergistically suppressing the basal activity. To design an inducible system, several cytoplasmic receptors were engineered by fusing chemical dimerizing domains (CID) with scTEVp parts. Upon the addition of DA, the proteolytic activity is restored, and the PH adducts are cleaved to afford de-shielded tTA. The addition of RAPA and ABA reconstituted the receptor’s proteolytic activity when the CID domains were integrated N’ terminally. The GID/GAI-based receptor was nonresponsive to GA, but this was overcome by ensuring the proper orientation of the receptor parts.

For ER-resident receptors, however, a different engineering approach was employed. In this compartment, the POI was retained and accumulated inside the ER by tagging its C’-terminus with a TEVp-cleavable KDEL sequence. The GA-regulated receptor again required the appropriate topological orientation, as in the cytoplasm, while the ABA-controlled receptor required specific mutagenesis to abolish detrimental PTMs: disulfide bridge formation and N-linked glycosylation. The same strategy, namely site-directed mutagenesis of disulfide-bond-forming residues, was employed to maintain the activity of cell-membrane-anchored OCAR receptors for regulated cell signaling. Since these OCAR receptors are among the few known Notch-based receptors regulated by soluble small ligands, we examined their mode of action. For this end, we co-cultured two cell populations, each expressing only one part of the receptor, in the absence/presence of the relevant inducer. As can be seen in Fig. 4c, ABA-regulated OCAR works in a cis-manner and both receptor parts are required for proper activation. However, RAPA- and GA-regulated OCARs required only the engineered part of the receptor to respond to their inducers (Fig. 5b, e). Although the precise molecular mechanism of RAPA- and GA-mediated OCAR activation remains elusive, we established that these synthetic receptors are functional and reliably trigger the orthogonal signaling cascade in the presence of functional γ-secretase (Fig. 6b, c).

The N308Q mutation in the ABI domain significantly improved the dimerization ability with PYL1 inside the ER when ABA was added (Fig. 3c). In addition, the two cysteine mutations C29S and C102S further enhanced its performance in this compartment (Fig. 3d). To better understand how these mutations rescued the receptor activity, we looked at the x-ray structure of PYL1/ABA/ABI complex^61^. Yin et al. have demonstrated that following ABA binding, PYL1 protein undergo a conformational change in the CL2 loop which enables efficient dimerization with the ABI domain. Furthermore, it has been shown that the interacting part of ABI contains a Mn^+2^-binding domain that participates in the interface interactions with ABA-bound PYL1 complex. Interestingly, we noticed that the D307 residue of ABI (referred as D413) participates in the formation of the Mn^+2^-containing domain, which is required for proper dimerization with PYL1-ABA. Therefore, it seems that glycosylation of adjacent N308 may disturb the formation of this domain and abolish its dimerization capability. The precise molecular mechanism underlying the effects of the C29S and C102S mutations is unclear. However, it seems likely that these mutations may play a role in preventing the formation of functionally harmful intra-/intermolecular disulfide bonds. To validate our hypothesis regarding N308Q mutation, and to establish how cysteine mutagenesis rescues ABI dimerization ability in the secretory pathway, further structural studies will be needed.

Structurally, Notch receptors are composed of a ligand-binding domain, negative regulatory region (NRR), and intracellular domain^55,62^. Following ligand binding, Notch receptor undergo a conformational change that triggers cleavage by membrane-located proteases (such as ADAM and γ-secretase). This process leads to liberation of the intracellular part of the receptor, which can be translocated to the nucleus to regulate gene expression. The NRR part is located between the ligand-binding domain and transmembrane part of Notch receptors and serves to prevent ligand-independent cleavage. This part of the receptor is composed of three cysteine-rich LIN-12-Notch repeats (LNRs) and a hetero-dimerization domain. In the OCAR design, we used the minimal Notch1 receptor core scaffold, which includes the NRR part. However, following their construction, initial evaluation showed that none of these receptors responds to its chemical activator. This led us to hypothesize that a functionally destructive intramolecular disulfide bond(s) between two distantly located cysteine residues in the dimerization domain and Notch1 core may be responsible for the lack of activity of the unmodified OCARs. Indeed, we saw that cysteine mutagenesis could restore the activity of the OCAR receptors activities, presumably by abolishing these functional harmful PTMs in the secretory pathway.

In summary, our engineered receptors are expected to be useful tools for fine-tuning orthogonal signaling in mammalian cells. We have provided a number of proof-of-concept applications and also characterized these synthetic constructs. The ER-retained receptors enable rapid secretion of POI within a few minutes using small molecules as a signal input. Such fast-responding systems could be utilized in cell-based therapy for diseases, such as diabetes, pain, and epilepsy, where the rapid release of therapeutics is required. The cell-membrane-anchored OCARs also enable different levels of signaling control. Due to their membrane location and their mechanism of action, OCAR systems possess an intrinsic off-switch, which could be used as a safety switch to shut down therapeutic cells’ gene circuits in the event of toxicity or malfunction. This would be especially important for inducers with a long half-life, which may remain in the blood circulation for several days after their discontinuation. In addition, we showed that ABA-regulated OCAR could be adjusted, through coiled-coil interactions, to enhanced SynNotch signaling during cell-cell communication. We think that the receptor architectures introduced here will find multiple applications in synthetic biology, basic science, and cell-based therapy.

## Methods

### Chemical reagents

Rapamycin (cat. no. 553210), gibberellic acid (cat. no. 48880), γ-secretase inhibitor LY411575 (cat. no. SML0506), FK506 (cat. no. 342500), and cycloheximide (cat. no. 01810) were purchased from Sigma-Aldrich, Buchs, Switzerland. (+)-Abscisic acid (Cat. No. 6554) was purchased from Tocris Bioscience, Bio-Techne AG, Switzerland. 4-Acetamido-4’-maleimidylstilbene-2,2’-disulfonic acid, disodium salt (AMS) (cat. no. A485) was purchased from Thermo Fisher Scientific, Reinach, Switzerland.

### Plasmid construction

Gene expression vectors were constructed either by using restriction enzymes (New England BioLabs, Ipswich, MA, USA) followed by ligation with T4 DNA ligase (New England BioLabs, cat. no. M0202L) or by Gibson assembly (New England BioLabs, cat. no. E2611L). For restriction enzyme-based cloning, digested plasmid backbones were dephosphorylated with Antarctic phosphatase before ligation (New England BioLabs, cat. no. M0289L). PCR reactions were performed using Q5 High-Fidelity DNA polymerase (New England BioLabs, cat. no. M0491L). For Gibson assembly, the PCR products were amplified using primers having 15–20 bp complementary sequences to each end of the linearized vector. Site-directed mutagenesis was performed using a Q5® Site-Directed Mutagenesis Kit (New England BioLabs, cat. no. E0552S). Detailed information about plasmid cloning is presented in Supplementary Tables S1 and S2. Plasmids were transformed and propagated in XL10-Gold® ultra-competent *Escherichia coli* (New England BioLabs, cat. no. C2992) and extracted using a plasmid mini-prep kit (Zymo Research, Irvine, CA, USA, cat. no. D4054) or a ZymoPURE II Plasmid Midiprep Kit (Zymo Research, cat. no. D4200).

### Cell culture

Human embryonic kidney cells (HEK-293T, ATCC: CRL-3216) were cultured in Dulbecco’s modified Eagle’s medium (DMEM; Thermo Fisher Scientific, cat. no. 10566016) supplemented with 10% fetal bovine serum (FBS; Sigma-Aldrich, cat. no. F7524, lot no. 022M3395) and penicillin (100 U)-streptomycin (100 µg) solution (Sigma-Aldrich, cat. no. P433) under a humidified atmosphere of 5% CO_2_ in air at 37 °C. Passaging of pre-confluent HEK-293T cultures was performed by trypsinization with 0.05% trypsin-EDTA (Life Technologies, Carlsbad, CA, USA; cat. no. 25300-054) for 5 min at 37 °C. Cells were transferred to 10 ml cell culture medium, and centrifuged for 1 min at 200 × g. The supernatant was discarded and the cells were resuspended in a fresh medium. Cell number and viability were quantified using an electric field multichannel cell-counting device (Casy® Cell Counter and Analyzer Model TT, Roche Diagnostics GmbH, Rotkreuz, Switzerland).

### Transient transfection

For plasmid transfection in a 96-well format, HEK-293T cells were seeded at a density of 50,000 cells per 1 cm^2^ in 100 μl medium for 24 h. 100 μl of serum and antibiotics-free minimum essential medium MEM (Thermo Fisher Scientific, cat. no. 11095080) containing a 1:5 DNA:PEI mixture (polyethylenimine, MW 40,000; Polysciences Inc., Warrington, FL, USA, cat. no. 24765) with a total DNA amount of 350 ng/cm^2^ was added dropwise to the cells, and the plate was incubated for 16 h.

### Pharmacological treatment

Unless stated otherwise, the culture medium of the transfected cells was replaced with 100 µl of fresh culture medium containing the indicated drugs and incubated for 24 h.

### SEAP quantification

SEAP levels in the cell culture medium were determined as follows: 20 µl of the culture supernatant was mixed with 80 µl ddH_2_O and heat-inactivated for 30 min at 65 °C. Then, 80 µl of 2x SEAP buffer (20 mM homoarginine, 1 mM MgCl_2_, 21% (v/v) diethanolamine, pH 9.8) and 20 µl of 120 mM para-nitrophenyl phosphate (Acros Organics, Geel, Belgium, cat. no. 128860100) solution in 2× SEAP buffer were added to each well, and the absorbance at 405 nm was measured at 37 °C using a Tecan M1000 plate reader (Tecan Group Ltd., Maennedorf, Switzerland). SEAP concentrations were calculated from a standard curve.

### Cell viability assay

Cell viability was evaluated using alamarBlue™ Cell Viability Reagent (Thermo Fisher, cat. no. DAL1025) according to the manufacturer’s instructions.

### Co-culture experiments

Receiver cells: HEK-293 cells were seeded in 6-well plates at a density of 0.4 × 10^6^ cells per well. The following day, cells were transfected with 2.5 µg total DNA. 0.4 µg reporter plasmid was used for all samples. Sender cells: HEK-293 cells were seeded in 10-cm plates at a density of 2 × 10^6^ cells per plate. On the following day, cells were transfected with 10 µg plasmid that encodes the relevant receptor/protein or with pcDNA3.1(+) as a negative control. 24 h following the transfection, sender and receiver cells were harvested and co-cultured in a 96-well plate by mixing 2 × 10^4^ receiver cells with 8 × 10^4^ sender cells (1:4 ratio). Drugs were added immediately, and samples were analyzed after 24 h.

### Western blotting

Cells were harvested, centrifuged at 1000 × g for 5 min, and washed twice in cold PBS. For cell lysis, RIPA buffer supplemented with protease and phosphatase inhibitor cocktail (Thermo Fisher, cat. no. A32963) was added to the cell pellet and the mixture was vortexed for 20 min at 4 °C. Lysates were cleared by centrifugation at 12,000 × g for 30 min at 4 °C. 5× reduced Laemmli sample buffer was added, boiled for 5 min at 95 °C, and loaded on SDS-PAGE. Protein quantification was performed using a Pierce™ BCA Protein Assay Kit (Thermo Fisher, cat. no. 23227). Following SDS-PAGE, gels were blotted onto PVDF membranes using a Biorad PowerPac^TM^. Blots were blocked with 10% skim milk in TBST buffer at room temperature for 1 h. The following primary antibodies were used (in 1:1000 dilution): monoclonal anti-FLAG® M2 antibody produced in mouse (Sigma, cat. no. F1804, clone M2), rabbit anti-β-actin (Cell Signaling, cat. no. 4970, clone 13E5). Secondary HRP-conjugated goat anti-rabbit (cat. no. 111-035-144, polyclonal) and anti-mouse (cat. no. 115-035-003, polyclonal) antibodies were purchased from Jackson Immunoresearch, West Grove, PA and used at a dilution of 1:10,000 dilution. PageRuler™ Plus Prestained Protein Ladder, 10–250 kDa (Thermo Fisher, cat. no. 26619) was used as protein molecular weight marker. Blots were developed using FUSION Pulse TS (cat. no. 37480003, Vilber, France), and images were analyzed using Adobe Illustrator software. All uncropped and unprocessed western blot images are provided in the Source Data file.

### AMS shift assay

Thiol alkylation of proteins using the sulfhydryl-specific reagent AMS was performed as described^63^. Briefly, the cleared lysates were treated with dithiothreitol (DTT) at 200 mM final concentration. Water was added to untreated samples. All samples were incubated for 5 min at 100 °C. Next, all samples were precipitated with 10% (w/v) trichloroacetic acid (TCA) for 30 min at 4 °C, and the precipitated proteins were recovered by centrifugation for 30 min at 14,000 g. Protein pellets were washed three times with 1 ml of ice-cold acetone followed by centrifugation for 15 min at 14,000 g. Protein pellets were air-dried and resuspended in 40 µl of an alkylating buffer (150 mM Tris–HCl (pH 7.5) and 2% SDS) containing/not containing 20 mM AMS and incubated for 2 h at 22 °C with moderate shaking. 10 µl of 5× sample buffer (containing DTT) was added to all samples, followed by boiling for 5 min. A 10 µl aliquot of each sample was subjected to SDS-PAGE.

### Cycloheximide chase experiment

To evaluate protein stability, cells were first transfected with the indicated expression vectors in a well of a 6-well plate. 24 h after transfection, cells were harvested, washed twice with PBS, and resuspended in cycloheximide-containing PBS (50 µg/ml). Cells were incubated at 37 °C and samples were collected at different time points. Lysates were prepared from the samples and subjected to SDS-PAGE.

### N-linked glycosylation analysis

Cell lysates were isolated and quantified as described before. Endoglycosidase treatment was performed using Endo-H (New England BioLabs, cat. no. P0702L) according to the manufacturer’s instructions.

### Figures

Figures were designed and assembled using Adobe Illustrator 2021 software.

### Statistics and reproducibility

The statistical significance of differences among groups was evaluated with a two-tailed, unpaired Student’s *t*-test using GraphPad Prism. Differences are considered statistically significant at *P* < 0.05. The statistical test used and the significance are reported in the figure legends. All the presented data has been independently repeated three times.

### Reporting summary

Further information on research design is available in the Nature Portfolio Reporting Summary linked to this article.

## Supplementary information

Supplementary Information

Reporting Summary

## Data Availability

The authors declare that all data generated in this study are provided within the paper and in the Supplementary Information/Source Data file. All vector information is provided in Supplementary Tables S1 and S2. Requests for materials should be made to the corresponding author. All plasmids generated in this study are available upon request. Source Data are provided with this paper.

## Source data

Source Data
