# Supplementary material for: Engineering receptors in the secretory pathway for orthogonal signalling control
Source: Nat Commun. 2022 Nov 29;13:7350. doi: 10.1038/s41467-022-35161-0 (PMC9708828; doi:10.1038/s41467-022-35161-0)
Supplement: Supplementary file 1 — Supplementary Information [file 41467_2022_35161_MOESM1_ESM.pdf]

Supplementary Material

Engineering Receptors in the Secretory Pathway for Orthogonal Signalling Control

Mohamed Mahameed[1], Pengli Wang[1], Shuai Xue[1], Martin Fussenegger[1,2,*]

[1] ETH Zürich, Department of Biosystems Science and Engineering, Mattenstrasse 26, 4058 Basel, Switzerland.

[2] University of Basel, Faculty of Life Science, Basel, Switzerland.

[*]Corresponding author. Email: fussenegger@bsse.ethz.ch

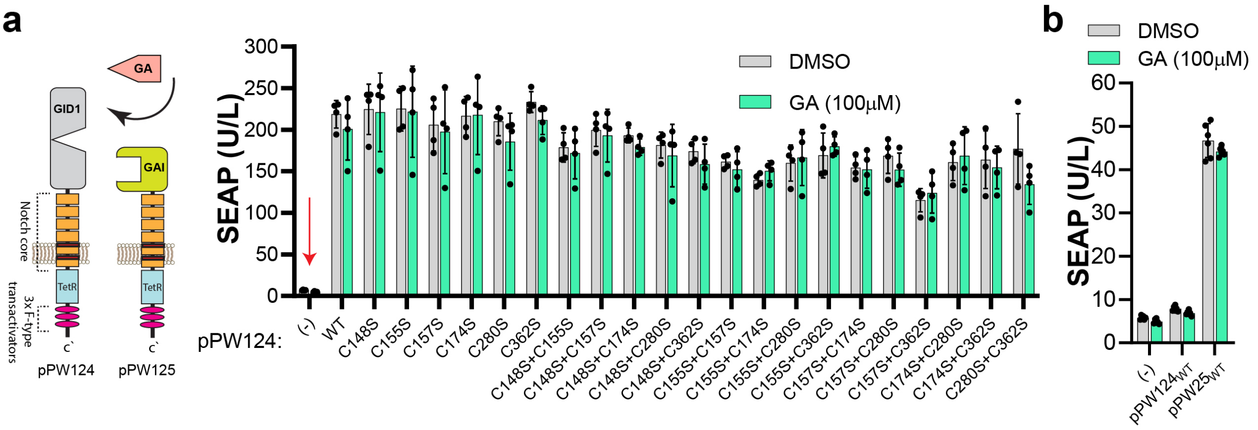

Fig. S1. a, Systematic cysteine (C) to serine (S) mutagenesis analysis of pPW124 (P_{PGK}-SS-GID1-Notch1$_{core}$-TetR-VP64-pA). HEK-293 cells were transfected with pPW125$_{WT}$ (P_{PGK}-SS-GAI$_{WT}$-Notch1$_{core}$-TetR-VP64-pA) and different C to S mutants of pPW124 (40 ng each). All samples were transfected with 20 ng of reporter plasmid pTS1017. SEAP levels were measured at 24 h after GA (100 μM) treatment. Data are presented as means ± s.d. of n = 4 biologically independent samples. **b,** SEAP levels in HEK-293 cells separately transfected with either pPW124$_{WT}$ or pPW125$_{WT}$ (40 ng each). All samples were transfected with 20 ng of reporter plasmid pTS1017. SEAP levels were quantified at 24 h after GA (100 μM) treatment. Data are presented as means ± s.d. of n = 6 biologically independent samples. ***P < 0.0001 was calculated using two-tailed, paired Student's t-test. Source data are provided as a Source Data file.

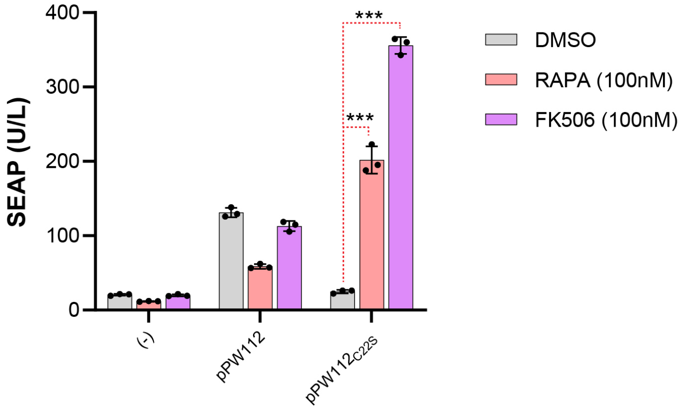

Fig. S2. SEAP levels in supernatants of HEK-293 cells transfected with pPW112 or pPW112$_{C22S}$ (40 ng each). All samples were transfected with 20 ng of reporter plasmid, pTS1017, which encodes tetO$_7$-driven SEAP ($P_{hCMV*-1}$-SEAP-pA$_{bGH}$; $P_{hCMV*-1}$, O_{tetO7}-$P_{hCMVmin}$). At 24 h after transfection, the culture medium was replaced with fresh medium containing either DMSO, RAPA (100 nM), or FK506 (100 nM). SEAP levels were measured at 24 h following the pharmacological treatments. Data are presented as means ± s.d. of n = 3 biologically independent samples. ***P < 0.0001 was calculated using two-tailed, paired Student's t-test. Source data are provided as a Source Data file.

Table S1

Plasmid	Information and Design	Reference
pcDNA3.1(+)	Mammalian expression vector.<br>(P_{hCMV}-MCS-pA_{bGH})	Life Technologies, Carlsbad, CA
pBMN DHFR(DD)-YFP	Expression plasmid used as a template for PCR amplification of folA dihydrofolate reductase destable domain (DD).	(Iwamoto et al., 2010)
pBS717	Tetracycline-inducible mammalian NLuc expression vector.<br>($P_{hCMV*-1}$-IgK-NLuc-pAbGH; $P_{hCMV*-1}$-NLuc-pA_{bGH})	(Stefanov et al., 2021)
pMM328	Vector encoding P_{hPGK}-driven expression of SEAP.<br>(P_{hPGK}-SEAP-pA_{bGH})	(Chassin et al., 2019)
pSLQ2812 pPB	Expression plasmid used as a template for PCR amplification of GID1 and GAI domains.	(Gao et al., 2016)
pVAMSyB Tier-1	Tier-1 mammalian expression vector.<br>(P_{hCMV}-MCS-pA_{bGH})	(Haellman et al., 2021)
pVAMSyB Tier-2	Tier-2 mammalian expression vector with multiple expression cassettes.<br>(MCS-pA_{bGH}- MCS- pA_{p36}-MCS- pA_{p9})	(Haellman et al., 2021)
SV-ABAactDA	Expression plasmid used as a template for PCR amplification of ABI and PYL1 domains.	(Liang et al., 2011)
pTS2405	Constitutive mammalian expression of intact cytoplasmic TEVp in Tier-1.<br>(P_{hCMV}-TEVp-pA_{bGH}).	Strittmatter et al., unpublished
pMMH75	Constitutive mammalian expression of DRD1 in Tier-1.<br>(P_{hCMV}-DRD1-pA_{bGH}).	Mahameed et al., unpublished
pMMH10	Constitutive mammalian expression of SEAP-TCS-KDEL.<br>(P_{hCMV}-SEAP-TCS-KDEL-pA_{bGH})	(Mahameed et al., 2022)
pMMH26	Constitutive mammalian expression vector for SS-FKBP-$(GGGGS)_2$-ss$TEVp_{1-118}$-KDEL.<br>(P_{hCMV}-SS-FKBP-$(GGGGS)_2$-ss$TEVp_{1-118}$-KDEL-pA_{bGH})	(Mahameed et al., 2022)
pMMH27	Constitutive mammalian expression vector for SS-FRB-ss$TEVp_{119-245}$-KDEL.<br>(P_{hCMV}-SS-FRB-ss$TEVp_{119-245}$-KDEL-pA_{bGH})	(Mahameed et al., 2022)
pMMH37	Constitutive mammalian expression vector for SS-FRB- ss$TEVp_{119-245}$-KDEL.<br>(P_{hPGK}-SS-FRB-ss$TEVp_{119-245}$-KDEL-pA_{bGH})	(Mahameed et al., 2022)
pMMH51	Constitutive mammalian expression vector for intact sec-TEVp-KDEL.<br>(P_{hCMV}-secTEVp-KDEL-pA_{bGH})	(Mahameed et al., 2022)
pMMH55	Constitutive mammalian expression vector for SS-FKBP-$(GGGGS)_2$-ssTEVp1-118-KDEL in Tier-2.<br>($P_{hEF1\alpha\text{-core}}$ - SS-FKBP-$(GGGGS)_2$-ss$TEVp_{1-118}$-KDEL-pA_{p9})	(Mahameed et al., 2022)

INS-2A-luciferase-2A-CD19-GFP	Constitutive mammalian expression vector for CD19.	(Ma et al., 2020)
HR_PGK_antiCD19_synNotch_TetRVP64	Lentiviral expression vector for constitutive expression of the anti-CD19 scFv-synNotch-TetRVP64 Receptor.	(Morsut et al., 2016)
pMM506	Constitutive mammalian expression vector of tTA in Tier-1.<br>(P_{hCMV}-tTA-pA_{bGH})	(Müller et al., 2017)
pTS1017	Tetracycline-responsive SEAP expression vector.<br>($P_{hCMV*-1}$-SEAP-pA_{bGH})	(Scheller et al., 2020)
pAna8	Vector encoding P_{hCMV}-driven expression of tTA-PH. PH domain was PCR amplified from PH-PLCD1-GFP (Addgene no. 51407) using the following primers: 5'-taagcagctagcggtggttctggtgactcgggccgggacttcctga and 5'-taagcaggatccgaagttctgcagctccttgaagctca. The PCR product was digested and ligated, through *Nhe*I/*Bam*HI, into pMM506.<br>(P_{hCMV}-tTA-PH-pA_{bGH})	Teixeira et al., unpublished
pAna21	Vector encoding P_{hPGK}-driven expression of tTA-PH. P_{hPGK} was excised from pMM328 and ligated into pAna8 through *Mlu*I/*Eco*RI restriction.<br>(P_{hPGK}-tTA-PH-pA_{bGH})	Teixeira et al., unpublished
pAna31	Vector encoding P_{hPGK}-driven expression of tTA-PH_{x2}. PH domain was PCR amplified from pAPT21 using the following primers: 5'-taagcaggatccgctagtgctagcggtggttc and 5'-taagtctagacaccggtgaagttctgcagctccttg. The resulted PCR was cloned into pAPT21 through *Bam*HI/*Xba*I.<br>(P_{hPGK}-tTA-PH_{x2}-pA_{bGH})	Teixeira et al., unpublished
PH-PLCD1-GFP	Expression plasmid used as a template for PCR amplification of PH domain.<br>(P_{hCMV}-GFP-PH-pA_{bGH})	(Várnai and Balla, 1998)
pMMH5	Constitutive mammalian expression vector encoding PH-tTA. PH domain was PCR amplified from PH-PLCD1-GFP (Addgene no. 51407) using the following primers: 5`-agaattcatggactcgggccgggacttcc and 5`-tactagtcttgtcatcgtcatccttgtagtcggcgaccggtggatccttcag-3`.The resulted PCR was cloned into pMM506 through *Eco*RI/*Spe*I.<br>(P_{hCMV}-PH-tTA-pA_{bGH})	This work
pMMH18	Constitutive mammalian expression vector encoding PH-tTA-PH_{x2}. PH domain was PCR amplified from pMMH5 using the following primers: 5`-agaattcatggactcgggccgggacttcc and 5`-tactagtcttgtcatcgtcatccttgtagtcggcgaccggtggatccttcag.The resulted PCR product was cloned into pAna31 through *Eco*RI/*Spe*I restriction enzymes.<br>(P_{hPGK}-PH-tTA-PH_{x2}-pA_{bGH})	This work
pMMH43	Constitutive mammalian expression vector encoding GAI-ssTEV$p_{119-245}$-KDEL. This construct was cloned by Gibson assembly of two fragments using *Eco*RI/*Hin*dIII-linearized pMM506. The first fragment encoding GAI was PCR amplified from pSLQ2812 pPB (Addgene no. 84240) using the primers: 5`-agctgttcgaagcggaattcatgctgctatccgtgccgctgctgctcggcctcctcggcctggccgtcgccatgaagagagatcatcatcatcat and 5`-	This work

	ggagaccatggagctcatggatttattaaggtcggtgagcatagaatc. The second fragment encoding ssTEVp$_{119-245}$-KDEL, was PCR amplified from pMMH27 using primers: 5`- aaatccatgagctccatggtctcc and 5` gcaggccggcctcaaagcttttacaactcgtccttagagccgcc. (P$_{hCMV}$-SS-GAI-ssTEVp$_{119-245}$-KDEL-pA$_{bGH}$).	
pMMH44	Constitutive mammalian expression vector encoding SS-PYL1-ssTEVp$_{119-245}$-KDEL. This construct was cloned by Gibson assembly of two fragments using *Eco*RI/*Hin*dIII linearized pMM506. The first fragment encoding PYL1 was PCR amplified from SV-ABAactDA (Addgene no. 38247) using primers: 5`- agctgttcgaagcggaattcatgctgctatccgtgccgctgctgctcggcctcctcggcctggccgtc gccggcgcgccaactcaagacgaattc and 5`- ggagaccatggagctcatggatttgttcatagcttcagtgatcgaagc. The second fragment encoding ssTEVp$_{119-245}$-KDEL, was PCR amplified from pMMH27 using primers: 5`- aaatccatgagctccatggtctcc and 5`-gcaggccggcctcaaagcttttacaactcgtccttagagccgcc. (P$_{hCMV}$- SS-PYL1-ssTEVp$_{119-245}$-KDEL-pA$_{bGH}$)	This work
pMMH45	Constitutive mammalian expression vector encoding SS-ABI-ssTEVp$_{1-118}$-KDEL. This construct was cloned by Gibson assembly of two fragments using *Eco*RI/*Hin*dIII linearized pMM506. The first fragment encoding ABI was PCR amplified from SV-ABAactDA (Addgene no. 38247) using primers: 5`- agctgttcgaagcggaattcatgctgctatccgtgccgctgctgctcggcctcctcggcctggccgtc gccacgcgtgtgcctttgtatggtttt and 5`- ctttccccttgcgcgccctgtgaacctccaccgccagaaccacctccgcccttcaaatcaaccaccac cacact. The second fragment encoding ssTEVp$_{1-118}$-KDEL, was PCR amplified from pMMH26 using priemrs: 5`- cagggcgcgcaaggggaaagcctg and 5`- gcaggccggcctcaaagcttttacaactcgtccttagagccgccggtctggaagttggtagtcacca g. (P$_{hCMV}$-SS-ABI-(GGGGS)$_2$-ssTEVp$_{1-118}$-KDEL-pA$_{bGH}$)	This work
pMMH46	Constitutive mammalian expression vector encoding SS-GID1-ssTEVp$_{1-118}$-KDEL. This construct was cloned by Gibson assembly of two fragments using *Eco*RI/*Hin*dIII linearized pMM506. First, GID1 was PCR amplified from pSLQ2812 pPB (Addgene no. 84240) using primers: 5`- agctgttcgaagcggaattcatgctgctatccgtgccgctgctgctcggcctcctcggcctggccgtc gccatggctgcgagcgatgaagttaat and 5`- ctttccccttgcgcgccctgtgaacctccaccgccagaaccacctccgccacattccgcgtttacaaa cgccga.The second fragment, ssTEVp$_{1-118}$-KDEL, was PCR amplified from pMMH26 using the following primers: 5`- cagggcgcgcaaggggaaagcctg and 5`- gcaggccggcctcaaagcttttacaactcgtccttagagccgccggtctggaagttggtagtcacca g.(P$_{hCMV}$-SS-ABI-(GGGGS)$_2$-ssTEVp$_{1-118}$-KDEL-pA$_{bGH}$)	This work
pMMH125a*	Constitutive mammalian expression vector encoding PH$_{x2}$-tTA-PH$_{x2}$. PH domain was amplified from PH-PLCD1-GFP (Addgene no. 51407) using primers: 5`- aaggatgacgatgacaagactagtatggactcgggccgggacttcctgaccctg and 5`- acttttatctaatctggaactagtagaaccaccagaaccaccggactgaaagtacaggttttcagaacc	This work

	accagaaccaccagaaccaccatccttcaggaagttctgcagctccttgaa. The resulting fragment was cloned into pMMH18 linearized with *Spe*I.<br>(P$_{hPGK}$-PH$_{x2}$-tTA-PH$_{x2}$-pA$_{bGH}$).	
pMMH125b*	Constitutive mammalian expression vector encoding PH$_{x2}$-TCS-tTA-TCS-PH$_{x2}$. This construct was cloned by combining three fragments by Gibson assembly using pMMH125a as a PCR template. The following primers were used to clone this vector:<br>PHx4-F-1 5`-Ggtatagggagaccgaattcatggactcgggccgggacttcctgaccctg and PHx4-R-1-5`-ggactgaaagtacaggttttcagaaccaccagaaccaccagaaccaccatccttcaggaagttctgcagctccttgaagct<br>PHx4F-2 5`-tgcgctgttaatcactttacttttatctaatctggaactagtagaaccaccagaaccaccggactgaaagtacaggttttc<br>PHx4-R-2 5`-ggactgaaagtacaggttttctgaacctccaccgccagatccgccacccccatattcatctattccaagggcatcggtaaa<br>PHx4-F-3 5` gtaatctggaacatcgtatgggtatgaacctccaccgccagaaccacctccgcccaattgggactgaaagtacaggttttc<br>PHx4-R-3 5`-atccctgcaggccggcctcaaagctttctagacaccggtgaagttctgcagctccttgaagctcatcttgttgtccttgtt. The resulting PCR fragments were cloned into *Eco*RI/*Hin*dIII digested pMMH18 by Gibson assembly.<br>(P$_{hPGK}$-PH$_{x2}$-TCS-tTA-TCS-PH$_{x2}$-pA$_{bGH}$).	This work
pMMH130	Mammalian P$_{hPGK}$-driven SS-GAI-Notch1$_{core}$ expression vector. GAI-Notch1$_{core}$ was amplified from pPW125 using the primers: 5`-ggtatagggagaccgaattcatgctgctatccgtgccgctgctgctcggc<br>and : 5`- tgcaggccggcctcaaagcttttacaacttttgtatacaaagttgccgccggcg. The resulted PCR was cloned, by Gibson assembly, to *Eco*RI/*Hin*dIII linearized pPW125.<br>(P$_{hPGK}$-SS-GAI$_{WT}$-Notch1$_{core}$-pA$_{bGH}$)	This work
pMMH139	Mammalian expression vector encoding FKBP- (GGGGS)$_2$-scTEVp$_{1-118}$. FKBP-(GGGGS)$_2$-scTEVp$_{1-118}$ was PCR amplified from pMMH26 using the primers: 5`-agctgttcgaagcggaattcatgggagtgcaggtggaaaccatctcccca and 5`-cctgcaggccggcctcaaagctt. The resulting PCR product was inserted by Gibson assembly into *EcoR*I/*Hind*III-linearized pMM506.<br>(P$_{hCMV}$-FKBP-(GGGGS)$_2$-scTEVp$_{1-118}$-pA$_{bGH}$)	This work
pMMH140	Mammalian expression vector encoding FRB-scTEVp$_{119-245}$. FRB-scTEVp$_{119-245}$ was PCR amplified from pMMH27 using the primers: 5`- agctgttcgaagcggaattcatgatcctctggcatgagatgtggcatgaa and 5`-cctgcaggccggcctcaaagctt. The resulting PCR product was inserted by Gibson assembly into *Eco*RI/*Hin*dIII linearized pMM506.<br>(P$_{hCMV}$-FRB-scTEVp$_{119-245}$-pA$_{bGH}$)	This work
pMMH171	Constitutive mammalian expression vector encoding P$_{hPGK}$-driven tTA expression. tTA was excised from pMM506 and cloned *Eco*RI/*Sbf*I into pMMH37.<br>(P$_{hPGK}$-tTA-pA$_{bGH}$)	This work

pMMH172	Constitutive mammalian expression vector encoding P_{hPGK}-driven PH-tTA expression. PH-tTA was excised from pMMH5 and cloned *Eco*RI/*Sbf*I into pMMH37.<br>(P_{hPGK}-PH-tTA-pA_{bGH})	This work
pMMH173	Constitutive mammalian expression vector encoding P_{hPGK}-driven PH-tTA-PH expression. PH-tTA-PH was PCR amplified from pMMH18 using the primers: 5`- gcccgagctcggtatagggagaccgaattc and 5`-caggccggcctcaaagcttctaaccagaaccaccgctagcactagcggatcc. the resulting PCR product was cloned *EcoR*I/*Hind*III into linearized pMMH37 by Gibson assembly.<br>(P_{hPGK}-PH-tTA-PH-pA_{bGH})	This work
pMMH174	Constitutive mammalian expression vector encoding P_{hPGK}-driven PH_{x2}-tTA expression. PH_{x2}-tTA was PCR amplified from pMMH125a using the primers: 5`- gcccgagctcggtatagggagaccgaattc and 5`-gcaggccggcctcaaagcttctaaccccccatattcatctattccaagggcatc. The resulting PCR product was cloned into *Eco*RI/*Hin*dIII linearized pMMH37 by Gibson assembly.<br>(P_{hPGK}-PH_{x2}-tTA-pA_{bGH})	This work
pMMH201	Constitutive mammalian expression vector encoding P_{hPGK}-driven Src_{MS}-TCS-tTA expression. tTA was PCR amplified from pMMH171 using the primers 5`-cggtatagggagaccgaattcatgggctccagcaagagtaaaccaaaggacccctcacagagaggtggaggttctgaaaacctgtactttcagtccggtggaggttccagattagataaaagtaaagtgattaacagcgcatta and 5`- cctgcaggccggcctcaaagctt. The resulting PCR product was cloned *Eco*RI/*Hin*dIII into linearized pMMH37 by Gibson assembly.<br>(P_{hPGK}-Src_{MS}-TCS-tTA-pA_{bGH})	This work
pMMH202	Constitutive mammalian expression vector encoding P_{hPGK}-driven ecDHFR(DD)-TCS-tTA expression. ecDHFR(DD) was PCR amplified from pBMN DHFR(DD)-YFP (Addgene no. 29325) using the primers: 5`- ggtatagggagaccgaattcatgatcagtctgattgcggcgttagcggtagattac and 5`-gttaatcactttacttttatctaatctggaactagttccacctccaccggactgaaagtacaggttttcagatccacctccaccccgctccagaatctcaaagcaatagctgtgagagtt.<br>The resulting PCR product was cloned *Eco*RI/*Spe*I into linearized pMMH171 by Gibson assembly.<br>(P_{hPGK}-ecDHFR(DD)-TCS-tTA-pA_{bGH})	This work
pMMH203	Constitutive mammalian expression vector encoding P_{hPGK}-driven DRD1-TCS-tTA expression. DRD1 was PCR amplified from pMMH75 using the primers: 5`-ggtatagggagaccgaattcatgcgcacattgaatacttcagcgatggacgga and 5`-tttacttttatctaatctggaactagtagttccacctccaccggactgaaagtacaggttttcagatccacctccaccagttggatgttgaccgttctgtgttattggttgaat.<br>The resulting PCR product was cloned *Eco*RI/*Spe*I into linearized pMMH171 through Gibson assembly.<br>(P_{hPGK}-DRD1-TCS-tTA-pA_{bGH}).	This work
pMMH204	Constitutive mammalian expression vector encoding P_{hPGK}-driven SS-ABI $_{C29S,C102S}$-Ex3-Notch1$_{core}$-TetR-VP64 expression. This construct was cloned through two fragments by Gibson assembly using *Eco*RI/*Hin*dIII linearized pMMH37. Two PCR products were amplified from pPW116$_{C29S,C102S}$ using the primers: 5`-	This work

	ggtatagggagaccgaattcatgctgctatccgtgccgctgctgctcggc ; 5`- ttccttctctagcgcagcgatttctttctccagggcggcaatttctgagccaccttcaaatcaaccaccaccacacttatgttgtc and 5`- gaaatcgctgcgctagagaaggaaatcgccgcactcgagaagggtgggggctcaatcctggactacagcttcacaggtggcgctggg ; 5`- cctgcaggccggcctcaaagctt (P_{hPGK}-SS-ABI$_{C29S,C102S}$-Ex3-Notch1$_{core}$-TetR-VP64-pA$_{bGH}$).	
pMMH205	Constitutive mammalian expression vector encoding P_{hPGK}-driven SS-ABI $_{C29S,C102S}$-Notch1$_{core}$-Ex3-TetR-VP64 expression. This construct was cloned through two fragments by Gibson assembly using *Eco*RI/*Hin*dIII linearized pMMH37. Two PCR products were amplified from pPW116$_{C29S,C102S}$ using the primers: 5`- ggtatagggagaccgaattcatgctgctatccgtgccgctgctgctcggc ; 5`- ttccttctctagcgcagcgatttctttctccagggcggcaatttctgagccacccaacttttgtatacaaagttgccgccggcgctt and 5`- gaaatcgctgcgctagagaaggaaatcgccgcactcgagaagggtgggggctcaatgtctagactggacaagagcaaagtcataaac ; 5`- cctgcaggccggcctcaaagctt (P_{hPGK}-SS-ABI$_{C29S,C102S}$-Notch1$_{core}$-Ex3-TetR-VP64-pA$_{bGH}$).	This work
pMMH206	Constitutive mammalian expression vector encoding P_{hPGK}-driven SS-PYL1-Ex3-Notch1$_{core}$-TetR-VP64 expression. This construct was cloned through two fragments by Gibson assembly using *Eco*RI/*Hin*dIII linearized pMMH37. Two PCR products were amplified from pPW86 using the primers: 5`- ggtatagggagaccgaattcatgctgctatccgtgccgctgctgctcggc ; 5`- ttccttctctagcgcagcgatttctttctccagggcggcaatttctgagccaccgttcatagcttcagtgatcgaagcaagtttctg and 5`- gaaatcgctgcgctagagaaggaaatcgccgcactcgagaagggtgggggctcaatcctggactacagcttcacaggtggcgctggg ; 5`- cctgcaggccggcctcaaagctt (P_{hPGK}-SS-PYL1-Ex3-Notch1$_{core}$-TetR-VP64-pA$_{bGH}$).	This work
pMMH207	Constitutive mammalian expression vector encoding P_{hPGK}-driven SS-PYL1-Notch1$_{core}$-Ex3-TetR-VP64 expression. This construct was cloned through two fragments by Gibson assembly using *Eco*RI/*Hin*dIII linearized pMMH37. Two PCR products were amplified from pPW86 using the primers: 5`- ggtatagggagaccgaattcatgctgctatccgtgccgctgctgctcggc ; 5`- ttccttctctagcgcagcgatttctttctccagggcggcaatttctgagccacccaacttttgtatacaaagttgccgccggcgctt and 5`- gaaatcgctgcgctagagaaggaaatcgccgcactcgagaagggtgggggctcaatgtctagactggacaagagcaaagtcataaac; 5`- cctgcaggccggcctcaaagctt (P_{hPGK}-SS-PYL1-Notch1$_{core}$-Ex3-TetR-VP64-pA$_{bGH}$).	This work
pMMH208	Constitutive mammalian expression vector encoding P_{hPGK}-driven SS-PYL1-Ex3-Notch1$_{core}$ expression. This construct was cloned through two fragments by Gibson assembly using *Eco*RI/*Hin*dIII linearized pMMH37. Two PCR products were amplified from pPW97 using the primers: 5`- ggtatagggagaccgaattcatgctgctatccgtgccgctgctgctcggc ; 5`- ttccttctctagcgcagcgatttctttctccagggcggcaatttctgagccaccgttcatagcttcagtgatcgaagcaagtttctg and 5`- gaaatcgctgcgctagagaaggaaatcgccgcactcgagaagggtgggggctcaatcctggactacagcttcacaggtggcgctggg; 5`- cctgcaggccggcctcaaagctt (P_{hPGK}-SS-PYL1-Ex3-Notch1$_{core}$-pA$_{bGH}$).	This work

pMMH209	Constitutive mammalian expression vector encoding P_{hPGK}-driven SS-PYL1-Notch1$_{core}$-Ex3 expression. This construct was cloned by Gibson assembly using *Eco*RI/*Hin*dIII linearized pMMH37. PCR product was amplified from pPW97 using the primers: 5`-ggtatagggagaccgaattcatgctgctatccgtgccgctgctgctcggc ; 5`-ccggcctcaaagcttttacttctcgagtgcggcgatttccttctctagcgcagcgatttctttctccagggcggcaatttctgagccacccaacttttgtatacaaagttgccgccggcg (P_{hPGK}-SS-PYL1-Notch1$_{core}$-Ex3-pA_{bGH}).	This work
pMMH210	Constitutive mammalian expression vector encoding P_{hPGK}-driven anti-CD19-Notch1$_{core}$-TetR-VP64 expression. This construct was cloned through two fragments by Gibson assembly using *Eco*RI/*Hin*dIII linearized pMMH37. The first PCR product was amplified from HR-PGK-antiCD19-synNotch-TetRVP64 (Addgene no.79126) using the primers: 5`- ggtatagggagaccgaattcatggcgctccctgtcaccgcactgcttcttccg; 5`- tgtgaagctgtagtccaggattgaactcactgtcactgacgtgccttgccccca. The second PCR product was amplified from pPW116$_{C29S,C102S}$ using the primers: 5`- atcctggactacagcttcacaggtggcgct; 5`-cctgcaggccggcctcaaagctt. (P_{hPGK}-SS-anti-CD19-Notch1$_{core}$-TetR-VP64-pA_{bGH}).	This work
pMMH211	Constitutive mammalian expression vector encoding P_{hPGK}-driven anti-CD19-Kx3-Notch1$_{core}$-TetR-VP64 expression. This construct was cloned through two fragments by Gibson assembly using *Eco*RI/*Hin*dIII linearized pMMH37. Two PCR products were amplified from pMMH210 using the following primers: 5`-ggtatagggagaccgaattcatggcgctccctgtcaccgcactgcttcttccg; 5`-tttctccttcagggcagcaatcttctccttcagggcagcaatctttgagccacctgaactcactgtcactgacgtgccttgccccca and 5`-aagattgctgccctgaaggagaaaatcgccgcgctcaaagagggtgggggctcaatcctggactacagcttcacaggtggcgctggg ; 5`- cctgcaggccggcctcaaagctt. (P_{hPGK}- anti-CD19-Kx3-Notch1$_{core}$-TetR-VP64-pA_{bGH}).	This work
pMMH212	Constitutive mammalian expression vector encoding P_{hPGK}-driven anti-CD19-Notch1$_{core}$-Kx3-TetR-VP64 expression. This construct was cloned through two fragments by Gibson assembly using *Eco*RI/*Hin*dIII linearized pMMH37. Two PCR products were amplified from pMMH210 using the primers: 5`-ggtatagggagaccgaattcatggcgctccctgtcaccgcactgcttcttccg; 5`-tttctccttcagggcagcaatcttctccttcagggcagcaatctttgagccacccaacttttgtatacaaagttgccgccggcgctt and 5`-aagattgctgccctgaaggagaaaatcgccgcgctcaaagagggtgggggctcaatgtctagactggacaagagcaaagtcataaac ; 5`- cctgcaggccggcctcaaagctt. (P_{hPGK}- anti-CD19-Notch1$_{core}$-Kx3-TetR-VP64-pA_{bGH}).	This work
pMMH230	Constitutive mammalian expression vector encoding P_{hPGK}-driven SS-FKBP-Notch1$_{core}$-TetR-VP64-2xFLAG expression. SS-FKBP-Notch1$_{core}$-TetR-VP64-2xFLAG was PCR amplified from pPW112 using the primers: 5`-ggtatagggagaccgaattcatgctgctatccgtgccgctgctgctcggc; 5`-tgcaggccggcctcaaagcttttacttgtcatcgtcgtccttgtagtcgcccttgtcatcgtcgtccttgtagtccccggggagcatatcaaggtcaaaatcgtc. The resulting PCR product was cloned by Gibson assembly into pMMH37 linearized by *Eco*RI/*Hin*dIII.	This work

	(P_{hPGK}-SS-FKBP-Notch1$_{core}$-TetR-VP64-2xFLAG-pA$_{bGH}$).	
pMMH231	Constitutive mammalian expression vector encoding P_{hPGK}-driven SS-FKBP$_{C22S}$-Notch1$_{core}$-TetR-VP64-2xFLAG expression. SS-FKBP$_{C22S}$-Notch1$_{core}$-TetR-VP64-2xFLAG was PCR amplified from pPW112$_{C22S}$ using the primers: 5`-ggtatagggagaccgaattcatgctgctatccgtgccgctgctgctcggc; 5`-tgcaggccggcctcaaagcttttacttgtcatcgtcgtccttgtagtcgccccttgtcatcgtcgtccttgtagtccccggggagcatatcaaggtcaaaatcgtc. The resulting PCR product was cloned by Gibson assembly into pMMH37 linearized by *Eco*RI/*Hin*dIII.<br>(P_{hPGK}-SS-FKBP$_{C22S}$-Notch1$_{core}$-TetR-VP64-2xFLAG -pA$_{bGH}$).	This work
pMMH232	Constitutive mammalian expression vector encoding P_{hPGK}-driven SS-GID1-Notch1$_{core}$-TetR-VP64-2xFLAG expression. SS-GID1-Notch1$_{core}$-TetR-VP64-2xFLAG was PCR amplified from pPW124 using the primers: 5`-ggtatagggagaccgaattcatgctgctatccgtgccgctgctgctcggc; 5`-tgcaggccggcctcaaagcttttacttgtcatcgtcgtccttgtagtcgccccttgtcatcgtcgtccttgtagtccccggggagcatatcaaggtcaaaatcgtc. The resulting PCR product was cloned by Gibson assembly into pMMH37 linearized by *Eco*RI/*Hin*dIII.<br>(P_{hPGK}-SS-GID1-Notch1$_{core}$-TetR-VP64-2xFLAG-pA$_{bGH}$).	This work
pMMH233	Constitutive mammalian expression vector encoding P_{hPGK}-driven SS-ABI $_{C29S,C102S}$ -Notch1$_{core}$-TetR-VP64-2xFLAG expression. SS-ABI$_{C29S,C102S}$ -Notch1$_{core}$-TetR-VP64-2xFLAG was PCR amplified from pPW116 $_{C29S,C102S}$ using the primers: 5`-ggtatagggagaccgaattcatgctgctatccgtgccgctgctgctcggc; 5`-tgcaggccggcctcaaagcttttacttgtcatcgtcgtccttgtagtcgccccttgtcatcgtcgtccttgtagtccccggggagcatatcaaggtcaaaatcgtc. The resulting PCR product was cloned by Gibson assembly into pMMH37 linearized by *Eco*RI/*Hin*dIII.<br>(P_{hPGK}-SS-ABI $_{C29S,C102S}$ -Notch1$_{core}$-TetR-VP64-2xFLAG-pA$_{bGH}$).	This work
pMMH234	Constitutive mammalian expression vector encoding P_{hPGK}-driven SS-ABI-Notch1$_{core}$-TetR-VP64-2xFLAG expression. SS-ABI-Notch1$_{core}$-TetR-VP64-2xFLAG was PCR amplified from pPW116 using the primers: 5`-ggtatagggagaccgaattcatgctgctatccgtgccgctgctgctcggc; 5`-tgcaggccggcctcaaagcttttacttgtcatcgtcgtccttgtagtcgccccttgtcatcgtcgtccttgtagtccccggggagcatatcaaggtcaaaatcgtc. The resulting PCR product was cloned by Gibson assembly into pMMH37 linearized by *Eco*RI/HindIII.<br>(P_{hPGK}-SS-ABI-Notch1$_{core}$-TetR-VP64-2xFLAG-pA$_{bGH}$).	This work
pMMH235	Constitutive mammalian expression vector encoding P_{hPGK}-driven SS-GID1$_{C155S,C157S,C362S}$-Notch1$_{core}$-TetR-VP64-2xFLAG expression. SS-GID1$_{C155S,C157S,C362S}$ -Notch1$_{core}$-TetR-VP64-2xFLAG was PCR amplified from pPW124 $_{C155S,C157S,C362S}$ using the primers: 5`-ggtatagggagaccgaattcatgctgctatccgtgccgctgctgctcggc; 5`-tgcaggccggcctcaaagcttttacttgtcatcgtcgtccttgtagtcgccccttgtcatcgtcgtccttgtagtccccggggagcatatcaaggtcaaaatcgtc. The resulting PCR product was cloned by Gibson assembly into pMMH37 linearized by *EcoR*I/*Hind*III.	This work

	(P$_{hPGK}$-SS-GID1$_{C155S,C157S,C362S}$-Notch1$_{core}$-TetR-VP64-2xFLAG-pA$_{bGH}$).	
pMMH241$_{C29S,C102S}$	Constitutive mammalian expression vector encoding P$_{hPGK}$-driven SS-ABI$_{C29S,C102S}$-Notch1$_{core}$ expression. SS-ABI$_{C29S,C102S}$-Notch1$_{core}$ was PCR amplified from pPW116$_{C29S,C102S}$ using the primers: 5`-ggtatagggagaccgaattcatgctgctatccgtgccgctgctgctcggc and 5`-tgcaggccggcctcaaagcttttacaacttttgtatacaaagttgccgccggcg. The resulting PCR fragment was cloned by Gibson assembly into pMMH37 linearized by *Eco*RI/*Hin*dIII. (P$_{hPGK}$-SS-ABI$_{C29S,C102S}$-Notch1$_{core}$-pA$_{bGH}$)	This work
pPW20	Constitutive mammalian expression vector encoding P$_{hCMV}$-driven ABI-(GGGGS)$_2$-ssTEVp$_{1-118}$-KDEL expression. ABI-(GGGGS)$_2$-ssTEVp$_{1-118}$-KDEL was PCR amplified from pMMH45 using the primers : 5`- aagcggaattcatgacgcgtgtgcctttgtatggtttt and 5`-gcctcaaagcttttacaactcgtccttagagccgcc and cloned *Eco*RI/*Hin*dIII into pMM506. (P$_{hCMV}$-ABI-(GGGGS)$_2$-ssTEVp$_{1-118}$-KDEL-pA$_{bGH}$)	This work
pPW21	Constitutive mammalian expression vector encoding P$_{hCMV}$-driven PYL1-ssTEVp$_{119-245}$-KDEL expression. PYL1-ssTEVp$_{119-245}$-KDEL was PCR amplified from pMMH44 using the primers : 5`-caccatgactagtggcgcgccaactcaagacgaattc and 5`-gcctcaaagcttttacaactcgtccttagagccgcc and cloned *Spe*I/*Hin*dIII into pMM506. (P$_{hCMV}$-PYL1-ssTEVp$_{119-245}$-KDEL-pA$_{bGH}$)	This work
pPW22	Constitutive mammalian expression vector encoding P$_{hCMV}$-driven GID1-(GGGGS)$_2$-ssTEVp$_{1-118}$-KDEL expression. GID1-(GGGGS)$_2$-ssTEVp$_{1-118}$-KDEL was PCR amplified from pMMH46 using the primers : 5`- aagcggaattcatggctgcgagcgatgaagttaat and 5`-gcctcaaagcttttacaactcgtccttagagccgcc and cloned *Eco*RI/*Hin*dIII into pMM506. (P$_{hCMV}$-GID1-(GGGGS)$_2$-ssTEVp$_{1-118}$-KDEL-pA$_{bGH}$)	This work
pPW23	Constitutive mammalian expression vector encoding P$_{hCMV}$-driven GAI-ssTEVp$_{119-245}$-KDEL expression. GAI-ssTEVp$_{119-245}$-KDEL was PCR amplified from pMMH43 using the primers : 5`-aagcggaattcatgaagagagagatcatcatcatcat and 5`-gcctcaaagcttttacaactcgtccttagagccgcc and cloned *Eco*RI/*Hin*dIII into pMM506. (P$_{hCMV}$-GAI-ssTEVp$_{119-245}$-KDEL-pA$_{bGH}$)	This work
pPW24	Constitutive mammalian expression vector encoding P$_{hCMV}$-driven ssTEVp$_{1-118}$-(GGGGS)$_2$-GID1 expression. First, GID1 was PCR amplified from pPW22 using the primers: 5`-atggctgcgagcgatgaagttaat and 5`- aggccggcctcaaagcttttaacattccgcgtttacaaacgc. ssTEVp$_{1-118}$-(GGGGS)$_2$ was PCR-amplified from pPW22 using the primers: 5`-ctgttcgaagcggaattcatgcagggcgcgcaaggggaaagc and 5`-ttcatcgctcgcagccattgaacctccaccgccagaaccacctccgcccaactcgtccttagagccgccgg. The resulting PCR products were cloned by Gibson assembly into pMM506 linearized by *Eco*RI/HindIII. (P$_{hCMV}$-ssTEVp$_{1-118}$-(GGGGS)$_2$-GID1-pA$_{bGH}$)	This work

pPW25	Constitutive mammalian expression vector encoding P_{hCMV}-driven ssTEVp$_{119-245}$-GAI expression. First, GAI was PCR-amplified from pPW23 using the primers: 5`- atgaagagagatcatcatcatcatcatcatcaagat and 5`- aggccggcctcaaagcttttaattaaggtcggtgagcatagaat. ssTEVp$_{119-245}$ was PCR-amplified from pPW23 using the primers: 5`- ctgttcgaagcggaattcatgaaatccatgagctccatggtctcc and 5`- atgatgatctctcttcattgaacctccaccgccagaaccacctccgcccaactcgtccttagagccgccttc. The resulting PCR products were cloned by Gibson assembly into pMM506 linearized by *Eco*RI/*Hin*dIII. (P_{hCMV}-ssTEVp$_{119-245}$-GAI-pA$_{bGH}$)	This work
pPW26	Constitutive mammalian expression vector encoding P_{hCMV}-driven SS-ssTEVp$_{1-118}$-(GGGGS)$_2$-GID1-KDEL expression. SS-ssTEVp$_{1-118}$-(GGGGS)$_2$-GID1-KDEL was PCR amplified from pPW24 using the primers: 5`- gttcgaagcggaattcatgctgctatccgtgccgctgctgctcggcctcctcggcctggccgtcgccatgcagggcgcgcaaggggaaagcctg and 5`- ccggcctcaaagcttttacaactcgtccttagaccccccacattccgcgtttacaaacgccgaaat. The resulting PCR products were cloned by Gibson assembly into pMM506 linearized by *Eco*RI/*Hin*dIII. (P_{hCMV}-SS-ssTEVp$_{1-118}$-(GGGGS)$_2$-GID1-KDEL-pA$_{bGH}$)	This work
pPW32	Constitutive mammalian expression vector encoding P_{hCMV}-driven ABI-(GGGGS)$_2$-ssTEVp$_{1-118}$-FLAG-KDEL expression. pPW20 was PCR amplified using the primers: 5`- gactacaaggacgacgatgacaagggcggctctaaggacgagttgtaaaagctttgaggc and 5`- ggtctggaagttggtagtcaccaggcagatgcgttcctc. The resulting PCR product was phosphorylated and ligated. (P_{hCMV}-ABI-(GGGGS)$_2$-ssTEVp$_{1-118}$-FLAG-KDEL-pA$_{bGH}$)	This work
pPW33	Constitutive mammalian expression vector encoding P_{hCMV}-driven PYL1-ssTEVp$_{119-245}$-FLAG-KDEL expression. pPW21 was PCR amplified using the primers: 5`- gactacaaggacgacgatgacaagggcggctctaaggacgagttgtaaaagctttgaggc and 5`- ttccaggccccccttcgttcatcagctgggtggcttcctt. The resulting PCR product was phosphorylated and ligated. (P_{hCMV}-PYL1-ssTEVp$_{119-245}$-FLAG-KDEL-pA$_{bGH}$)	This work
pPW34	Constitutive mammalian expression vector encoding P_{hCMV}-driven SS-ABI-(GGGGS)$_2$-ssTEVp$_{1-118}$-FLAG-KDEL expression. pMMH45 was PCR amplified using the primers: 5`- gactacaaggacgacgatgacaagggcggctctaaggacgagttgtaaaagctttgaggc and 5`- ggtctggaagttggtagtcaccaggcagatgcgttcctc. The resulting PCR product was phosphorylated and ligated. (P_{hCMV}-SS-ABI-(GGGGS)$_2$-ssTEVp$_{1-118}$-FLAG-KDEL-pA$_{bGH}$)	This work
pPW35	Constitutive mammalian expression vector encoding P_{hCMV}-driven SS-PYL1-ssTEVp$_{119-245}$-FLAG-KDEL expression. pMMH44 was PCR amplified using the primers: 5`- gactacaaggacgacgatgacaagggcggctctaaggacgagttgtaaaagctttgaggc and 5`- ttccaggccccccttcgttcatcagctgggtggcttcctt. The resulting PCR product was phosphorylated and ligated. (P_{hCMV}-SS-PYL1-ssTEVp$_{119-245}$-FLAG-KDEL-pA$_{bGH}$)	This work
pPW86	Constitutive mammalian expression vector encoding P_{hPGK}-driven SS-PYL1-Notch1$_{core}$-TetR-VP64 expression. PYL1 was PCR amplified	This work

	from pMMH44 using the primers: 5`-ggtatagggagaccgaattcatgctgctatccgtgccgctgctgctcggc and 5`-gtgaagctgtagtccaggatgttcatagcttcagtgatcgaagcaagttt. Notch1$_{core}$ was PCR amplified from pHR_PGK_antiCD19_synNotch_TetRVP64 (Addgene no.79126) using the primers: 5`-atcctggactacagcttcacaggtggcgct and 5`-caggccggcctcaaagcttttacccggggagcatgtcaaggtcaaaatcgtc. Both fragments were cloned by Gibson assembly into pMMH37 linearized by *Eco*RI/*Hin*dIII.<br>(P_{hPGK}-SS-PYL1-Notch1$_{core}$-TetR-VP64-pA$_{bGH}$)	
pPW97	Constitutive mammalian expression vector encoding P_{hPGK}-driven SS-PYL1-Notch1$_{core}$ expression. SS-PYL1-Notch1$_{core}$ was PCR amplified from pPW86 using the primers: 5`-ggtatagggagaccgaattcatgctgctatccgtgccgctgctgctcggc and 5`-tgcaggccggcctcaaagcttttacaacttttgtatacaaagttgccgccggcg. The resulted PCR fragment was cloned by Gibson assembly into pMMH37 linearized by *Eco*RI/*Hin*dIII.<br>(P_{hPGK}-SS-PYL1-Notch1$_{core}$-pA$_{bGH}$)	This work
pPW108	Constitutive mammalian expression vector encoding P_{hPGK}-driven SS-FKBP-(GGGS)$_2$-Notch1$_{core}$-TetR-VP64 expression. SS-FKBP-(GGGS)$_2$ was PCR amplified from pMMH55 using the primers: 5`-ggtatagggagaccgaattcatgctgctatccgtgccgctgctgctcggc and 5`-ccgccagaaccacctccgccttccagttttagaagctccacatcgaagac. Notch1$_{core}$ was PCR amplified from pPW86 using the primers: 5`-ggcggaggtggttctggcggtggagg and 5`- gcaggccggcctcaaagcttttacc. Both fragments were cloned by Gibson assembly into pMMH37 linearized by *Eco*RI/*Hin*dIII.<br>(P_{hPGK}-SS-FKBP-(GGGS)$_2$-Notch1$_{core}$-TetR-VP64-pA$_{bGH}$)	This work
pPW109	Constitutive mammalian expression vector encoding P_{hPGK}-driven SS-FRB-(GGGS)$_2$-Notch1$_{core}$-TetR-VP64 expression. SS-FRB-(GGGS)$_2$ was PCR amplified from pMMH37 using the primers: 5`-ggtatagggagaccgaattcatgctgctatccgtgccgctgctgctcggc and 5`-ccgccagaaccacctccgcccttt gagattcgtcggaacacatgataata. Notch1$_{core}$ was PCR amplified from pPW86 using the primers: 5`-ggcggaggtggttctggcggtggagg and 5`- gcaggccggcctcaaagcttttacc. Both fragments were cloned by Gibson assembly into pMMH37 linearized by *Eco*RI/*Hin*dIII.<br>(P_{hPGK}-SS-FRB-(GGGS)$_2$-Notch1$_{core}$-TetR-VP64-pA$_{bGH}$)	This work
pPW112	Constitutive mammalian expression vector encoding P_{hPGK}-driven SS-FKBP-Notch1$_{core}$-TetR-VP64 expression. SS-FKBP was PCR amplified from pMMH55 using the primers: 5`-ggtatagggagaccgaattcatgctgctatccgtgccgctgctgctcggc and 5`-gtgaagctgtagtccaggatttccagttttagaagctccacatcgaagac. Notch1$_{core}$-TetR-VP64 was PCR amplified from pPW86 using the primers: 5`-atcctggactacagcttcacaggtggcgct and 5`-gcaggccggcctcaaagcttttaccc. Both fragments were cloned by Gibson assembly into pMMH37 linearized by EcoRI/*Hin*dIII.<br>(P_{hPGK}-SS-FKBP-Notch1$_{core}$-TetR-VP64-pA$_{bGH}$)	This work

pPW113	Constitutive mammalian expression vector encoding P_{hPGK}-driven SS-FRB-Notch1$_{core}$-TetR-VP64 expression. SS-FRB was PCR amplified from pMMH37 using the primers: 5`-ggtatagggagaccgaattcatgctgctatccgtgccgctgctgctcggc and 5`-gtgaagctgtagtccaggatctttgagattcgtcggaacacatgataata. Notch1$_{core}$ was PCR amplified from pPW86 using the primers: 5`-atcctggactacagcttcacaggtggcgc and 5`- gcaggccggcctcaaagcttttaccc. Both fragments were cloned by Gibson assembly into pMMH37 linearized by *Eco*RI/*Hin*dIII. (P_{hPGK}-SS-FRB-Notch1$_{core}$-TetR-VP64-pA$_{bGH}$)	This work
pPW116	Constitutive mammalian expression vector encoding P_{hPGK}-driven SS-ABI-Notch1$_{core}$-TetR-VP64 expression. ABI was PCR amplified from pMMH45 using the primers: 5`-ggtatagggagaccgaattcatgctgctatccgtgccgctgctgctcggc and 5`-gtgaagctgtagtccaggatcttcaaatcaaccaccaccacacttatgtt. Notch1$_{core}$ was PCR amplified from pHR_PGK_antiCD19_synNotch_TetRVP64 (Addgene no.79126) using the primers: 5`-atcctggactacagcttcacaggtggcgct and 5`- gcaggccggcctcaaagcttttacc. Both fragments were cloned by Gibson assembly into pMMH37 linearized by *Eco*RI /*Hin*dIII. (P_{hPGK}-SS-ABI-Notch1$_{core}$-TetR-VP64-pA$_{bGH}$)	This work
pPW124	Constitutive mammalian expression vector encoding P_{hPGK}-driven SS-GID1-Notch1$_{core}$-TetR-VP64 expression. SS-GID1 was PCR amplified from pPW29 using the primers: 5`-ggtatagggagaccgaattcatgctgctatccgtgccgctgctgctcggc and 5`-gtgaagctgtagtccaggatacattccgcgtttacaaacgccgaaatct. Notch1$_{core}$-TetR-VP64 was PCR amplified from pPW86 using the primers: 5`-atcctggactacagcttcacaggtggcgct and 5`-gcaggccggcctcaaagcttttaccc. Both fragments were cloned by Gibson assembly into pMMH37 linearized by *Eco*RI/*Hin*dIII. (P_{hPGK}-SS-GID1-Notch1$_{core}$-TetR-VP64 -pA$_{bGH}$)	This work
pPW125	Constitutive mammalian expression vector encoding P_{hPGK}-driven SS-GAI-Notch1$_{core}$-TetR-VP64 expression. SS-GAI was PCR amplified from pPW28 using the primers: 5`-ggtatagggagaccgaattcatgctgctatccgtgccgctgctgctcggc and 5`-gtgaagctgtagtccaggatattaaggtcggtgagcatagaatcaagcca. Notch1$_{core}$ was PCR amplified from pPW86 using the primers: 5`-atcctggactacagcttcacaggtggcgc and 5`- gcaggccggcctcaaagcttttaccc. Both fragments were cloned by Gibson assembly into pMMH37 linearized by *Eco*RI/*Hin*dIII. (P_{hPGK}-SS-GAI-Notch1$_{core}$-TetR-VP64-pA$_{bGH}$)	This work

Primer pairs used for PCR amplification : The forward primer is shown first, followed by the reverse primer. Restriction sites and homology regions for Gibson assembly are underlined.

Abbreviations: **ABI**, abscisic acid receptor; **CD19,** cluster of differentiation 19; **DRD1,** dopamine receptor D_1; **ecDHFR(DD)**, Escherichia coli dihydrofolate reductase destabilizing domain; **GFP**, green-fluorescent protein; **FKBP**, FK506 binding protein ; **FRB**, FKBP-rapamycin binding; **GAI**, gibberellin insensitive protein; **GID1**, gibberellin insensitive dwarf1; **KDEL**, Lysine, Aspartic acid, Glutamic acid, Leucine; **KLD**, kinase, ligase, and DpnI; **MCS**, multiple cloning site; **MS,** myristylation signal; **NLuc**, nanoluc luciferase reporter gene; **O_{tetO7}**, heptameric TetR-specific operator; **pA_{bGH}**, polyadenylation signal from the bovine growth hormone; **PCR**,

polymerase chain reaction; **PH**, PLCD1 pleckstrin homology domain; **P_{hCMV}**, human cytomegalovirus immediate early promoter; **$P_{hCMVmin}$**, minimal version of P_{hCMV}; **$P_{hCMV*-1}$**, tetracycline-responsive promoter; **P_{hPGK}**, human 3-phosphoglycerate kinase promoter ; **PYL1**, pyrabactin resistance (PYR)/PYR1-like; **scFv**, single-chain variable fragment; **scTEVP**, split cytoplasmic tobacco etch virus protease; **SEAP**, human placental secreted alkaline phosphatase; **sec-TEVp**, secretory tobacco etch virus protease; **Src**, proto-oncogene tyrosine-protein kinase Src; **SS**, secretion signal; **ssTEVP**- split secretory tobacco etch virus protease; **TCS**, TEVp cleavage site; **TetR**, *Escherichia coli Tn*10-derived tetracycline-dependent repressor; **tTA**, tetracycline-dependent transactivator (TetR-VP16);**VP64**, transcriptional activator composed of four tandem copies of VP16 (Herpes Simplex Viral Protein 16).

Site-directed mutagenesis

Site-directed mutagenesis was performed using the Q5® Site-Directed Mutagenesis Kit (New England BioLabs, cat. no. E0552S) according to the manufacturer's instructions. Primers used during site-directed mutagenesis are presented in Table S2. Inserted mutations are underlined and shown in red. In cases where more than one mutation was required, we used the corresponding primers consecutively, unless otherwise stated (see note **).

Table S2

Plasmid	Mutation	Forward primer (5\`-3\`)	Reverse primer (5\`-3\`)
pMMH45	C29S	tttacttcgattagtggaagaagacctgagatgg	accatacaaaggcacacgcgtggc
	C82S	tagcgaactatagtagagagaggatgcatttgg	cctgagaaccgccatggccgtcgtaaac
	C102S	cgatgctcagcgatggtgatacgtgg	gtttctccttagctatctcctccgccaaag
	C153S	tcgtcgctaactgcggtgactc	agatgtgagacgggaaaacaacggc
	N308Q	gaagcaaagaccagataagtgtggtggtggttgatttg	ctctctgtatcgccagctttgacaaatactcagccg
pMMH44	C45S	ggtaacggccgtagctcatctctcctag	gagttggtacgtgtggaactcggcg
	C92S	gagtgggaagcacgcgcgacg	gcatctcgaaatcttcactcacgttacagc
	N81Q	gctgtcaggtgagtgaagatttcgagatgcgag	ttttgatgaagtgtttgtaaatctgtggcctatcg
	N105Q	ggcgcagacgtctcgagagagattagatctg	ggtaatccacttatcacgttcacgtcgcg
	N197Q	ctatgcagaaatccatgagctccatggtctccg	cttcagtgatcgaagcaagtttctgaagattcaatc
pPW108/112	C22S	agcgtggtgcactacaccgggatgcttgaa	ggtctggccgcgcttggggaaggtgcgccc
pPW109/113	C65S	agcaggaagtacatgaaatcagggaatgtc	ccactcttgggcctccattaaatctcgacc
pPW124	C148S	tctcgcaggcttgttggtttgtgcaagtgt	aagagtatcgtagatggcactgtttgcaga
	C155S	tccaagtgtgttgttgtctctgtgaattat	caaaccaacaagcctgcgacaaagagtatc
	C157S	tctgttgttgtctctgtgaattatcggcgt	cttgcacaaaccaacaagcctgcgacaaag
	C174S	tctgcttatgatgatggttggattgctctt	agggtatggattctctggtgcacgccgata
	C280S	tctaatccgtttagcccgagagggaaaagc	cgctggatgctctctatcttctccctcggg
	C362S	tctatcctggactacagcttcacaggtggc	ttccgcgtttacaaacgccgaaatctcatc
	C148S+C155S**	tctcgcaggcttgttggtttgtccaagtgt	aagagtatcgtagatggcactgtttgcaga
	C148S+C157S**	tctcgcaggcttgttggtttgtgcaagtct	aagagtatcgtagatggcactgtttgcaga

Notes

* These plasmids tend to recombine in the bacteria during their amplification. To prevent unwanted recombination reactions, these constructs were cultured at 30 °C instead of 37 °C.
** In these constructs, the target cysteine codons are close to each other and cannot be consecutively mutated with the original primers. We therefore designed new primers.

Supplementary References

1. Chassin, H., Müller, M., Tigges, M., Scheller, L., Lang, M., and Fussenegger, M. (2019). A modular degron library for synthetic circuits in mammalian cells. Nat. Commun. *10*.
2. Gao, Y., Xiong, X., Wong, S., Charles, E.J., Lim, W.A., and Qi, L.S. (2016). Complex transcriptional modulation with orthogonal and inducible dCas9 regulators. Nat. Methods *13*, 1043–1049.
3. Haellman, V., Strittmatter, T., Bertschi, A., Stücheli, P., and Fussenegger, M. (2021). A versatile plasmid architecture for mammalian synthetic biology (VAMSyB). Metab. Eng. *66*, 41–50.
4. Iwamoto, M., Björklund, T., Lundberg, C., Kirik, D., and Wandless, T.J. (2010). A general chemical method to regulate protein stability in the mammalian central nervous system. Chem. Biol. *17*, 981–988.
5. Liang, F. Sen, Ho, W.Q., and Crabtree, G.R. (2011). Engineering the ABA Plant stress pathway for regulation of induced proximity. Sci. Signal. *4*, 1–10.
6. Ma, H., Jeppesen, J.F., and Jaenisch, R. (2020). Human T Cells Expressing a CD19 CAR-T Receptor Provide Insights into Mechanisms of Human CD19-Positive β Cell Destruction. Cell Reports Med. *1*, 100097.
7. Mahameed, M., Xue, S., Stefanov, B.A., Hamri, G.C. El, and Fussenegger, M. (2022). Engineering a Rapid Insulin Release System Controlled By Oral Drug Administration. Adv. Sci. *9*, 1–10.
8. Morsut, L., Roybal, K.T., Xiong, X., Gordley, R.M., Coyle, S.M., Thomson, M., and Lim, W.A. (2016). Engineering Customized Cell Sensing and Response Behaviors Using Synthetic Notch Receptors. Cell *164*, 780–791.
9. Müller, M., Ausländer, S., Spinnler, A., Ausländer, D., Sikorski, J., Folcher, M., and Fussenegger, M. (2017). Designed cell consortia as fragrance-programmable analog-to-digital converters. Nat. Chem. Biol. *13*, 309–316.
10. Scheller, L., Schmollack, M., Bertschi, A., Mansouri, M., Saxena, P., and Fussenegger, M. (2020). Phosphoregulated orthogonal signal transduction in mammalian cells. Nat. Commun. *11*.
11. Stefanov, B.A., Teixeira, A.P., Mansouri, M., Bertschi, A., Krawczyk, K., Hamri, G.C. El, Xue, S., and Fussenegger, M. (2021). Genetically Encoded Protein Thermometer Enables Precise Electrothermal Control of Transgene Expression. Adv. Sci. *2101813*, 1–12.
12. Várnai, P., and Balla, T. (1998). Visualization of phosphoinositides that bind pleckstrin homology domains: Calcium- and agonist-induced dynamic changes and relationship to myo-[3H]inositol-labeled phosphoinositide pools. J. Cell Biol. *143*, 501–510.
