# Supplementary material for: Engineering receptors in the secretory pathway for orthogonal signalling control
Source: Nat Commun. 2022 Nov 29;13:7350. doi: 10.1038/s41467-022-35161-0 (PMC9708828; doi:10.1038/s41467-022-35161-0)
Supplement: Supplementary file 2 — Reporting Summary [file 41467_2022_35161_MOESM2_ESM.pdf]

nature research

Corresponding author(s): Martin Fussenegger

Last updated by author(s): Nov 17, 2022

# Reporting Summary

Nature Research wishes to improve the reproducibility of the work that we publish. This form provides structure for consistency and transparency in reporting. For further information on Nature Research policies, see our Editorial Policies and the Editorial Policy Checklist.

## Statistics

For all statistical analyses, confirm that the following items are present in the figure legend, table legend, main text, or Methods section.

| n/a | Confirmed | |
|---|---|---|
| ☐ | ☒ | The exact sample size ($n$) for each experimental group/condition, given as a discrete number and unit of measurement |
| ☐ | ☒ | A statement on whether measurements were taken from distinct samples or whether the same sample was measured repeatedly |
| ☐ | ☒ | The statistical test(s) used AND whether they are one- or two-sided<br>*Only common tests should be described solely by name; describe more complex techniques in the Methods section.* |
| ☒ | ☐ | A description of all covariates tested |
| ☐ | ☒ | A description of any assumptions or corrections, such as tests of normality and adjustment for multiple comparisons |
| ☐ | ☒ | A full description of the statistical parameters including central tendency (e.g. means) or other basic estimates (e.g. regression coefficient) AND variation (e.g. standard deviation) or associated estimates of uncertainty (e.g. confidence intervals) |
| ☐ | ☒ | For null hypothesis testing, the test statistic (e.g. $F$, $t$, $r$) with confidence intervals, effect sizes, degrees of freedom and $P$ value noted<br>*Give P values as exact values whenever suitable.* |
| ☒ | ☐ | For Bayesian analysis, information on the choice of priors and Markov chain Monte Carlo settings |
| ☒ | ☐ | For hierarchical and complex designs, identification of the appropriate level for tests and full reporting of outcomes |
| ☒ | ☐ | Estimates of effect sizes (e.g. Cohen's $d$, Pearson's $r$), indicating how they were calculated |

*Our web collection on statistics for biologists contains articles on many of the points above.*

## Software and code

Policy information about availability of computer code

| | |
|---|---|
| Data collection | Absorbance data were collected using TECAN AG, Maennedorf, Switzerland. Western blot images were developed using FUSION Pulse TS (cat. no.37480003, Vilber, France). |
| Data analysis | GraphPad Prism 8 and adobe illustrator 2021. |

For manuscripts utilizing custom algorithms or software that are central to the research but not yet described in published literature, software must be made available to editors and reviewers. We strongly encourage code deposition in a community repository (e.g. GitHub). See the Nature Research guidelines for submitting code & software for further information.

## Data

Policy information about availability of data

All manuscripts must include a data availability statement. This statement should provide the following information, where applicable:

- Accession codes, unique identifiers, or web links for publicly available datasets
- A list of figures that have associated raw data
- A description of any restrictions on data availability

The authors declare that all data generated in this study are provided within the paper and in the Supplementary Information/Source Data file. All vector information is provided in Supplementary Table 1 and Table 2. Requests for materials should be made to the corresponding author. All plasmids generated in this study are available upon request. Any additional relevant data are available from the authors upon reasonable request. Source data are provided with this paper.

# Field-specific reporting

Please select the one below that is the best fit for your research. If you are not sure, read the appropriate sections before making your selection.

- [x] Life sciences
- [ ] Behavioural & social sciences
- [ ] Ecological, evolutionary & environmental sciences

For a reference copy of the document with all sections, see nature.com/documents/nr-reporting-summary-flat.pdf

# Life sciences study design

All studies must disclose on these points even when the disclosure is negative.

| | |
|---|---|
| Sample size | No statistical methods were used to predetermine sample size. However, based on our previous experiments with similar settings, when we used the same sample number of control and treated group and assuming type I error =0.05 and the probability of a type II error=0.20, we required at least 2.6 samples for each group to detect 50% change between groups means. Therefore, we used at least 3 replicates for each group in this study. |
| Data exclusions | All data was included. |
| Replication | Attempts at replication were successful. Replication times are detailed in "Statistics and Reproducibility" section. |
| Randomization | Samples were randomly allocated into different experimental groups. |
| Blinding | The investigators were not blinded to allocation during experiments and outcome assessment. Blinding was not possible as the same investigator processed the experiments and analyzed the data. |

# Reporting for specific materials, systems and methods

We require information from authors about some types of materials, experimental systems and methods used in many studies. Here, indicate whether each material, system or method listed is relevant to your study. If you are not sure if a list item applies to your research, read the appropriate section before selecting a response.

## Materials & experimental systems

| n/a | Involved in the study | |
|---|---|---|
| [ ] | [x] | Antibodies |
| [ ] | [x] | Eukaryotic cell lines |
| [x] | [ ] | Palaeontology and archaeology |
| [x] | [ ] | Animals and other organisms |
| [x] | [ ] | Human research participants |
| [x] | [ ] | Clinical data |
| [x] | [ ] | Dual use research of concern |

## Methods

| n/a | Involved in the study | |
|---|---|---|
| [x] | [ ] | ChIP-seq |
| [x] | [ ] | Flow cytometry |
| [x] | [ ] | MRI-based neuroimaging |

## Antibodies

| | |
|---|---|
| Antibodies used | The following primary antibodies were used in this study (in 1:1000 dilution): monoclonal anti-FLAG® M2 antibody produced in mouse (Sigma, cat. no. F1804, clone M2), rabbit anti β-actin (Cell Signaling, cat. no. 4970, clone 13E5). Secondary HRP-conjugated goat anti-rabbit (Code: 111-035-144, polyclonal) and anti-mouse (Code: 115-035-003, polyclonal) antibodies were purchased from Jackson Immunoresearch, West Grove, PA and were used in 1:10,000 dilution. |
| Validation | Anti-FLAG M2 antibody was validated by the absence of bands when untransfected cells lysate was immunoblotted.<br>Rabbit anti β-actin antibody was validated by loading different amounts of cell lysate. A corelation between the lysate amount and band intensity in the correct protein size was noticed.<br>Both secondary antibodies were validated by the absence of bands/signal in immunoblot that has not been exposed to primary antibody. |

## Eukaryotic cell lines

Policy information about cell lines

| | |
|---|---|
| Cell line source(s) | Human embryonic kidney cells (HEK293T, ATCC: CRL-3216). |
| Authentication | Cell were authenticated by ATCC. All the phenotype of cell lines was frequently checked and controlled by microscopy. |
| Mycoplasma contamination | HEK293T cells were tested frequently for mycoplasma and confirmed as negative. |

| | |
|---|---|
| Commonly misidentified lines (See ICLAC register) | No commonly misidentified cell lines were used in the study. |

## Animals and other organisms

Policy information about studies involving animals; ARRIVE guidelines recommended for reporting animal research

| | |
|---|---|
| Laboratory animals | The study did not involve animal experiments. |
| Wild animals | The study did not involve wild animals. |
| Field-collected samples | The study did not involve field-collected samples. |
| Ethics oversight | The study did not involve animal experiments. |

Note that full information on the approval of the study protocol must also be provided in the manuscript.
